# Integrated causal inference, kidney transcriptomics, and experimental validation identify ChREBP (*MLXIPL*) as a driver of maladaptive metabolic remodeling in diabetic kidney disease

**DOI:** 10.3389/fendo.2026.1809567

**Published:** 2026-04-15

**Authors:** Mingliang Liu, Shihang Chen, Shi Wu, Bei Sun, Liming Chen

**Affiliations:** 1 School of Medicine, Nankai University, Tianjin, China; 2 NHC Key Laboratory of Hormones and Development, Tianjin Key Laboratory of Metabolic Diseases, Chu Hsien-I Memorial Hospital & Tianjin Institute of Endocrinology, Tianjin Medical University, Tianjin, China

**Keywords:** albuminuria, ChREBP, diabetic kidney disease, mendelian randomization, metabolic remodeling, MLXIPL

## Abstract

**Background:**

Diabetic kidney disease (DKD) remains a leading cause of end-stage renal disease despite advances in glucose-, blood pressure-, and albuminuria-lowering therapies. The glucose-responsive transcription factor carbohydrate response element-binding protein (ChREBP; encoded by *MLXIPL*) regulates glycolytic–lipogenic programs, yet its causal contribution to renal injury is challenging to disentangle in advanced DKD, where bulk kidney transcriptomes reflect tissue remodeling and cellular compositional shifts.

**Methods:**

We integrated two-sample Mendelian randomization (MR), kidney transcriptomic stratification, network analyses, and experimental validation. MR used blood cis-eQTL instruments for *MLXIPL* to estimate causal effects on type 2 diabetes (T2D) and urinary albumin-to-creatinine ratio (UACR), including a non-diabetic UACR stratum. In kidney transcriptomics (GSE30529), we evaluated remodeling-related confounding and applied within-DKD, median-based *MLXIPL*-high/low stratification for GSEA/GSVA and functional/network inference. Key observations were validated in db/db mice and primary proximal tubular epithelial cells (PTECs) exposed to high glucose with matched osmotic control.

**Results:**

Genetically predicted higher *MLXIPL* expression was associated with increased T2D risk across multiple phenotype definitions and with higher UACR, including replication in non-diabetic individuals. Within DKD, *MLXIPL* heterogeneity tracked metabolic programs by GSEA and divergent pathway activity by GSVA, while signatures related to profibrotic and proliferative remodeling were concomitantly enriched in the low-*MLXIPL* subgroup. Network analyses positioned *MLXIPL*/ChREBP within a dense metabolic interaction and regulatory landscape. Experimentally, ChREBP and *Mlxipl* were increased in db/db kidneys and induced by high glucose in PTECs, accompanied by coordinated upregulation of lipogenic targets (*Acly, Acaca, Fasn, Srebf1*) and an inverse relationship with *Ppargc1b*.

**Conclusions:**

Integrating genetic inference, confounding-aware kidney transcriptomics, network biology, and experimental validation, our study supports *MLXIPL*/ChREBP as a pathogenic nutrient-sensing node linking diabetes susceptibility to renal injury and maladaptive metabolic remodeling in DKD, providing a mechanistic rationale for targeting this axis to mitigate residual renal risk.

## 1 Introduction

Diabetic kidney disease (DKD) is a leading cause of end-stage renal disease and remains a major driver of morbidity and healthcare burden worldwide (1, 2). Although contemporary therapies targeting glycemia, blood pressure, and albuminuria have improved outcomes, many patients continue to experience progressive loss of renal function, underscoring a persistent “residual risk” and the urgent need for mechanism-based targets beyond conventional metabolic control (3–5). DKD is increasingly recognized as a disease of coupled metabolic stress and maladaptive tissue remodeling, in which nutrient overload, mitochondrial dysfunction, and lipotoxicity intersect with inflammatory and fibrotic signaling to accelerate tubular injury and interstitial fibrosis (6–9).

A central challenge in the field is identifying upstream transcriptional regulators that translate systemic nutrient excess into pathogenic metabolic rewiring specifically within the kidney (10). MLX interacting protein-like (*MLXIPL*), also known as carbohydrate response element-binding protein (ChREBP), is a glucose-responsive transcription factor that serves as a master regulator of glycolytic–lipogenic gene programs and lipid handling (11, 12). While dysregulated *MLXIPL*/ChREBP signaling has been implicated in systemic insulin resistance and ectopic lipid accumulation in metabolic organs, such as the liver and adipose tissue (13–15), its causal contribution to diabetes susceptibility and renal injury, as well as its associated molecular programs within diseased kidney tissue, remain incompletely defined (16). Notably, prior experimental studies have implicated ChREBP in diabetic kidney injury. Li et al. demonstrated that inducible podocyte-specific ChREBP knockdown ameliorated biochemical and histological manifestations of DKD in *db/db* mice, whereas Chen et al. showed that ChREBP deficiency attenuated diabetes-associated renal apoptosis and stress responses in diabetic models (17, 18). These findings provide important experimental support for a pathogenic role of ChREBP in diabetic renal injury. Nevertheless, to strengthen causal inference and assess its translational relevance in human disease, complementary evidence from large-scale human genetics and kidney transcriptomic analyses is still needed. Furthermore, mechanistic inference from bulk kidney transcriptomics is complicated by advanced DKD-associated remodeling and cellular compositional shifts (19). These confounding factors can obscure kidney-intrinsic metabolic signals, necessitating integrative strategies that go beyond simple case–control expression comparisons (20, 21). The clinical benefits of sodium-glucose cotransporter 2 inhibitors (SGLT2i) in slowing DKD progression further support the translational relevance of targeting renal nutrient-sensing and metabolic stress pathways, although whether these effects intersect directly with MLXIPL/ChREBP signaling remains to be clarified (22, 23).

To bridge these gaps, we integrated genetic causal inference with kidney transcriptomic and network analyses, followed by experimental validation. We applied two-sample Mendelian randomization (MR) using blood cis-eQTL instruments to test the causal effect of genetically predicted *MLXIPL* expression on type 2 diabetes (T2D) risk and renal damage, indexed by the urinary albumin-to-creatinine ratio (UACR) (24–26). And then we leveraged a DKD kidney transcriptomic cohort and implemented a within-DKD stratification strategy to resolve *MLXIPL*-linked transcriptional programs with minimized compositional bias (27), interrogating pathway activity using complementary enrichment approaches (GSEA/GSVA) and functional annotation (28, 29). We placed *MLXIPL* in a mechanistic context using protein–protein interaction mapping and multi-layer regulatory network inference. Finally, we validated key observations in diabetic db/db mouse kidneys and in primary proximal tubular epithelial cells (PTECs) exposed to high glucose. Collectively, this integrated framework aims to clarify the pathogenic relevance of *MLXIPL*/ChREBP and to define its associated metabolic programs in the diabetic kidney, providing a rationale for targeting nutrient-sensing transcriptional pathways in DKD.

## 2 Methods

### 2.1 Study design and overall workflow

This study integrated three complementary approaches to investigate the role of *MLXIPL*/ChREBP in diabetes-related outcomes and renal injury: two-sample Mendelian randomization (MR), bioinformatics analyses of public kidney transcriptomes, and experimental validation in diabetic mice and primary proximal tubular epithelial cells (PTECs).

### 2.2 Mendelian randomization analysis

#### 2.2.1 MR design and assumptions

A two-sample MR design was used. MR inference relies on three assumptions: (1) genetic variants are robustly associated with the exposure (*MLXIPL* expression); (2) variants are independent of confounders; and (3) variants influence outcomes only through the exposure (no horizontal pleiotropy) (24).

#### 2.2.2 Exposure data (genetic instruments for *MLXIPL* expression)

Summary statistics for SNP–*MLXIPL* expression associations were obtained from the eQTLGen Consortium cis-eQTL meta-analysis of 31,684 whole-blood samples of European ancestry (25). *Cis*-acting variants mapped to *MLXIPL* (chromosome 7) were extracted. Whole blood eQTLs were used as proxies for systemic regulation of *MLXIPL* expression.

#### 2.2.3 Outcome data sources and stratification rationale

Type 2 diabetes (T2D). Summary statistics were obtained from FinnGen (Release 10) (30). To assess robustness across phenotype definitions, six related endpoints were analyzed, including broad “Diabetes mellitus” (finn-b-E4_DIABETES), strict “Type 2 diabetes excluding T1D” (finn-b-E4_DM2_STRICT), and “Insulin-treated diabetes” (finn-b-KELA_DIAB_INSUL).

Renal damage (UACR). Summary statistics for urinary albumin-to-creatinine ratio (UACR) were obtained from the CKDGen Consortium (26). To explore whether *MLXIPL*-related renal injury may reflect hyperglycemia-mediated effects or potentially diabetes-independent mechanisms, we adopted a two-step strategy: (i) discovery analysis in the largest available dataset representing the overall population (ieu-a-1107; *N* = 54,450), followed by (ii) validation in a subset of non-diabetic individuals (ieu-a-1101) to evaluate whether associations persisted in a non-diabetic setting.

#### 2.2.4 Instrument selection, harmonization, and MR estimation

Variants associated with *MLXIPL* expression at genome-wide significance (P < 5×10^−8^) were eligible instruments. Linkage disequilibrium (LD) clumping was performed using a 10,000 kb window and r^2^ < 0.001 with the 1000 Genomes European reference panel (31). Instrument strength was evaluated using the F-statistic (F = β^2^/SE^2^); variants with F > 10 were considered strong instruments. Exposure and outcome datasets were harmonized to align effect alleles; palindromic SNPs with intermediate allele frequencies were excluded. For the UACR analyses, SNP retention was determined by variant availability after harmonization with the CKDGen outcome datasets, rather than by selecting variants with the largest exposure effect sizes or the strongest T2D associations.

The inverse-variance weighted (IVW) method was used as the primary estimator (32). For binary outcomes (T2D), effects are reported as odds ratios (OR) with 95% confidence intervals (CI); for UACR, effects are reported as beta coefficients with 95% CI.

#### 2.2.5 Sensitivity analyses and software

Robustness was assessed using weighted median and MR-Egger methods (33, 34). Directional pleiotropy was evaluated by the MR-Egger intercept test, and heterogeneity was assessed using Cochran’s Q statistic. Leave-one-out analyses were performed to evaluate whether results were driven by single instruments. As an additional tissue-relevance check, the lead blood cis-eQTL instruments were cross-referenced in GTEx v10 Kidney Cortex eQTL data by mapping the primary SNP instruments to kidney cortex variant-level associations for *MLXIPL*. MR analyses were conducted using TwoSampleMR (v0.5.6) in R (v4.4.3) (35). Two-sided P < 0.05 was considered statistically significant.

### 2.3 Bioinformatics and transcriptomic analyses

#### 2.3.1 Data acquisition and preprocessing

The microarray dataset GSE30529 (platform GPL571) was downloaded from the Gene Expression Omnibus (36, 37), and the specific information is shown in **Supplementary Table 1**. Raw CEL files were imported into R and preprocessed using standard microarray workflows, including background correction and quantile normalization (38), followed by probe re-annotation based on the platform annotation. Expression values were log_2_-transformed for downstream analyses.

#### 2.3.2 Differential expression and *MLXIPL*-based stratification

Differential expression between DKD and control renal tissues was assessed using the limma framework. To characterize *MLXIPL*-associated programs within DKD, DKD samples were stratified into *MLXIPL*-high and *MLXIPL*-low groups using the median *MLXIPL* expression as the cutoff, and differential expression between these subgroups was evaluated using limma. For visualization in the volcano plot, genes were displayed using a nominal P value threshold of < 0.05 without applying an additional absolute fold-change cutoff; genes were classified as upregulated or downregulated according to the sign of logFC. Where applicable, P values were adjusted using the Benjamini–Hochberg procedure.

#### 2.3.3 GSEA, GSVA, and functional enrichment

To identify *MLXIPL*-associated pathways, GSEA was performed using clusterProfiler with MSigDB curated gene sets (C2). Genes were ranked based on statistics from the *MLXIPL*-high vs *MLXIPL*-low comparison, and pathways with FDR < 0.25 were considered significantly enriched (28, 39, 40).

GSVA was performed using MSigDB Hallmark gene sets to compute pathway activity scores per sample (29, 40). Differences in GSVA scores between *MLXIPL*-high and *MLXIPL*-low DKD samples were evaluated using the Wilcoxon rank-sum test.

Functional enrichment of *MLXIPL*-associated differentially expressed genes was conducted for Gene Ontology (GO) and KEGG pathways using clusterProfiler. Multiple testing was controlled using the Benjamini–Hochberg method, and adjusted P < 0.05 was considered significant (39, 41, 42).

#### 2.3.4 Network construction

A high-confidence protein–protein interaction (PPI) network was constructed using STRING with a stringent confidence threshold, and additional functionally related genes were retrieved using GeneMANIA (43, 44). Networks were visualized in Cytoscape. Putative TF–*MLXIPL*, miRNA–*MLXIPL*, RNA-binding protein (RBP)–*MLXIPL*, and chemical/drug–*MLXIPL* relationships were compiled from ChIPBase, ENCORI/starBase, and the Comparative Toxicogenomics Database (CTD) using evidence-based filters as implemented in the analyses (45–47). These networks were interpreted as in silico, hypothesis-generating regulatory candidates.

#### 2.3.5 Immune infiltration analysis

Relative fractions of 22 immune cell subsets in renal tissues were estimated using CIBERSORT with the LM22 reference matrix and 1,000 permutations (20). Differences between DKD and control samples were assessed using the Wilcoxon rank-sum test. Associations between *MLXIPL* expression and immune cell fractions were evaluated using Spearman correlation, and immune landscapes were visualized using bar plots, box plots, and heatmaps.

#### 2.3.6 Additional analyses of bulk-tissue *MLXIPL* abundance and compositional features

To further evaluate whether bulk-tissue *MLXIPL* abundance in GSE30529 was influenced by tissue composition, additional correlation and multivariable regression analyses were performed. Tubular integrity, fibrosis, immune, and ChREBP target signature scores were calculated for each sample using predefined gene sets. The tubular integrity signature included *AQP1, LRP2, CUBN, SLC34A1, SLC5A2*, and *ALDOB*; the fibrosis signature included *COL1A1, COL3A1, FN1, ACTA2, TGFB1, VIM*, and *POSTN*; the immune signature included *PTPRC, CD68, CCL2, IL6, ITGAM*, and *HLA-DRA*; and the ChREBP target signature included *ACLY, ACACA, FASN, SCD, DGAT1*, and *TXNIP*. For each gene set, a sample-level score was defined as the mean z-scored expression of the included genes. Spearman’s rank correlation analysis was used to assess the associations between *MLXIPL* expression and these signature scores. Multivariable linear regression models were then fitted with *MLXIPL* expression as the dependent variable and DKD status as the primary independent variable, with sequential adjustment for tubular integrity, fibrosis, and immune signatures. Residualized *MLXIPL* expression after adjustment for these compositional features was also examined.

#### 2.3.7 Software and patient/public involvement

Bioinformatics analyses were performed in R (v4.4.3). Unless otherwise specified, two-sided P < 0.05 was considered statistically significant. Figures were generated using ggplot2 and pheatmap.

Patients and the public were not involved in the design, conduct, reporting, or dissemination plans of this research. This work is a secondary analysis of publicly available, de-identified transcriptomic data.

### 2.4 Experimental validation

#### 2.4.1 Animals and kidney tissue processing

Male *db/db* and *db/m* mice were used and sacrificed at 20 weeks of age. Mice were anesthetized with isoflurane and euthanized by cervical dislocation. Kidneys were collected immediately; tissue for RNA/protein extraction was snap-frozen, and tissue for histology was fixed in 4% paraformaldehyde, paraffin-embedded, and sectioned at 4 μm. All procedures were approved by the institutional animal ethics committee (Approval No.DXBYY-IACUC-2024034).

#### 2.4.2 Primary PTEC isolation, culture, and treatments

Primary proximal tubular epithelial cells (PTECs) were isolated from C57BL/6 mice by collagenase digestion followed by filtration to enrich tubular fractions. Cells were cultured in DMEM/F12 supplemented with fetal bovine serum at 37 °C with 5% CO_2_.

Cells were treated for 48 h under the following conditions:

Normal glucose (NG): 5.5 mM D-glucose;
High glucose (HG): NG medium supplemented with additional D-glucose to reach 25 mM (i.e., +19.5 mM);
Osmotic control: NG medium supplemented with 19.5 mM mannitol, matching the osmolarity increase of the HG condition.

#### 2.4.3 RNA extraction and qRT–PCR

Total RNA was extracted using TRIzol reagent. Reverse transcription was performed using an abm cDNA synthesis kit following the manufacturer’s instructions. Quantitative PCR was performed using SYBR Green chemistry. 18S was used as the internal control, and relative expression was calculated using the 2^−^ΔΔCt method. Primer sequences are provided in the **Supplementary Table 2**.

#### 2.4.4 Western blotting

Protein lysates were prepared using RIPA buffer supplemented with protease/phosphatase inhibitors and PMSF, followed by quantification using a BCA assay. Equal amounts of protein were subjected to SDS–PAGE and immunoblotting. ChREBP was detected using a primary antibody from Novus Biologicals (NB400-135; WB dilution 1:1000). β-actin served as the loading control. Signals were detected by ECL, and band intensities were quantified using ImageJ. *MLXIPL*/ChREBP protein was analyzed without distinguishing isoforms.

#### 2.4.5 Immunohistochemistry and immunofluorescence

For IHC, paraffin sections were deparaffinized and rehydrated, followed by antigen retrieval in Tris–EDTA buffer. Sections were blocked with 1% BSA and incubated with anti-ChREBP antibody (Novus Biologicals, NB400-135; IHC dilution 1:200), followed by appropriate secondary antibodies and chromogenic development.

For IF, PTECs were incubated with anti-ChREBP antibody (Novus Biologicals, NB400-135; IF dilution 1:50), followed by fluorescent secondary antibodies and DAPI counterstaining. Representative images were acquired by microscopy; quantitative image analysis was not performed.

#### 2.4.6 Correlation and statistical analysis

Pearson correlation analyses were performed using kidney tissues from 12 *db/m* and 12 *db/db* mice. Data are presented as mean ± SD. Two-group comparisons were performed using a two-tailed Student’s t-test. Statistical analyses were conducted using GraphPad Prism and R. Two-sided P < 0.05 was considered statistically significant.

## 3 Results

### 3.1 Genetic instruments for *MLXIPL* expression and harmonization

We identified *cis*-acting expression quantitative trait loci (*cis*-eQTLs) for *MLXIPL* using the eQTLGen Consortium meta-analysis (31,684 whole-blood samples). Following linkage disequilibrium (LD) clumping (P < 5×10^−8^, r^2^ < 0.001, window 10,000 kb), four independent SNPs located in the *MLXIPL cis*-region were retained as instrumental variables (**Table 1**: rs17145738, rs3812316, rs3812318, and rs7805165). The F-statistics for these instruments ranged from 2,131 to 2,962, exceeding the conventional threshold of (F > 10).

**Table 1:** Genetic instruments for *MLXIPL* expression derived from eQTLGen.

SNP	chr	pos	effect allele	other allele	eaf	beta	se	pval	F statistic
rs17145738	7	73082302	C	T	0.25	0.554	0.012	<0.001	2131.361
rs3812316	7	73095699	C	T	0.28	0.762	0.014	<0.001	2962.469
rs3812318	7	73095710	G	A	0.29	0.65	0.013	<0.001	2500.000
rs7805165	7	73108922	C	T	0.26	0.63	0.013	<0.001	2348.521

SNP, single-nucleotide polymorphism; EA, effect allele; OA, other allele; EAF, effect allele frequency; SE, standard error.

For the primary MR analyses of T2D outcomes in FinnGen, all four SNPs were harmonized and utilized. For the renal trait analyses involving UACR (CKDGen), variant coverage in the outcome datasets limited the analysis to two independent instruments (rs17145738 and rs7805165). These two SNPs were applied to MR analyses in both the discovery (overall population) and validation (non-diabetic) cohorts. As an additional tissue-level cross-reference, we examined the lead blood cis-eQTL instruments in GTEx v10 Kidney Cortex eQTL data. Two of the four primary SNPs (rs17145738 and rs3812316) were identifiable in the kidney cortex dataset and showed directionally consistent positive effects on *MLXIPL* expression (slope = 0.169 and 0.165, respectively), although neither reached nominal significance (P = 0.202 and 0.201, respectively) (**Supplementary Table 3**).

### 3.2 Causal association of genetically predicted *MLXIPL* expression with type 2 diabetes risk

**Figure 1:**
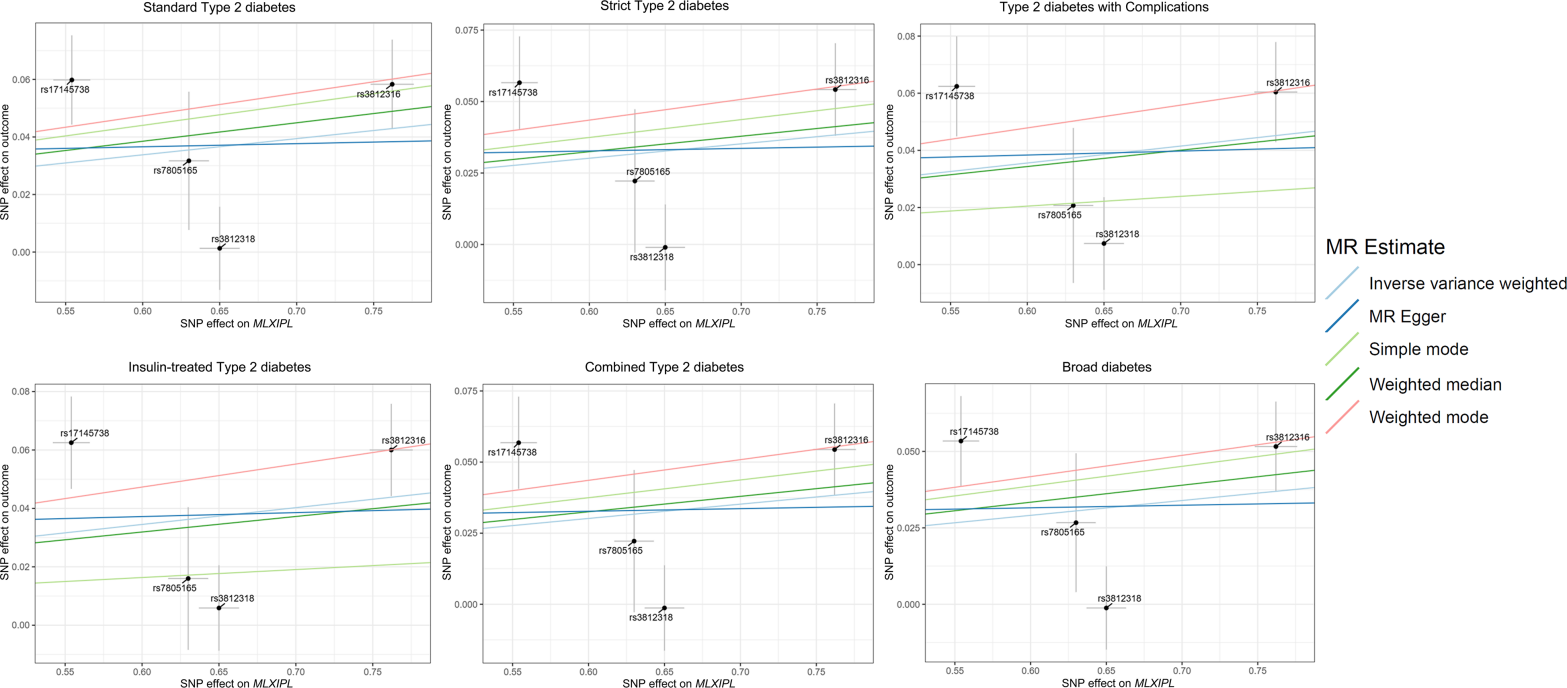
Mendelian randomization scatter plots illustrating the association between genetically predicted *MLXIPL* expression and Type 2 Diabetes across six phenotype definitions. Black points represent individual SNP instruments and are labeled by rsID. Analyses were based on four cis-eQTL instruments, all of which were strong instruments (all F-statistics > 10). The fitted lines represent the primary random-effects IVW estimates. Heterogeneity and pleiotropy statistics are reported in **Supplementary Tables 4**, **5**.

**Figure 2:**
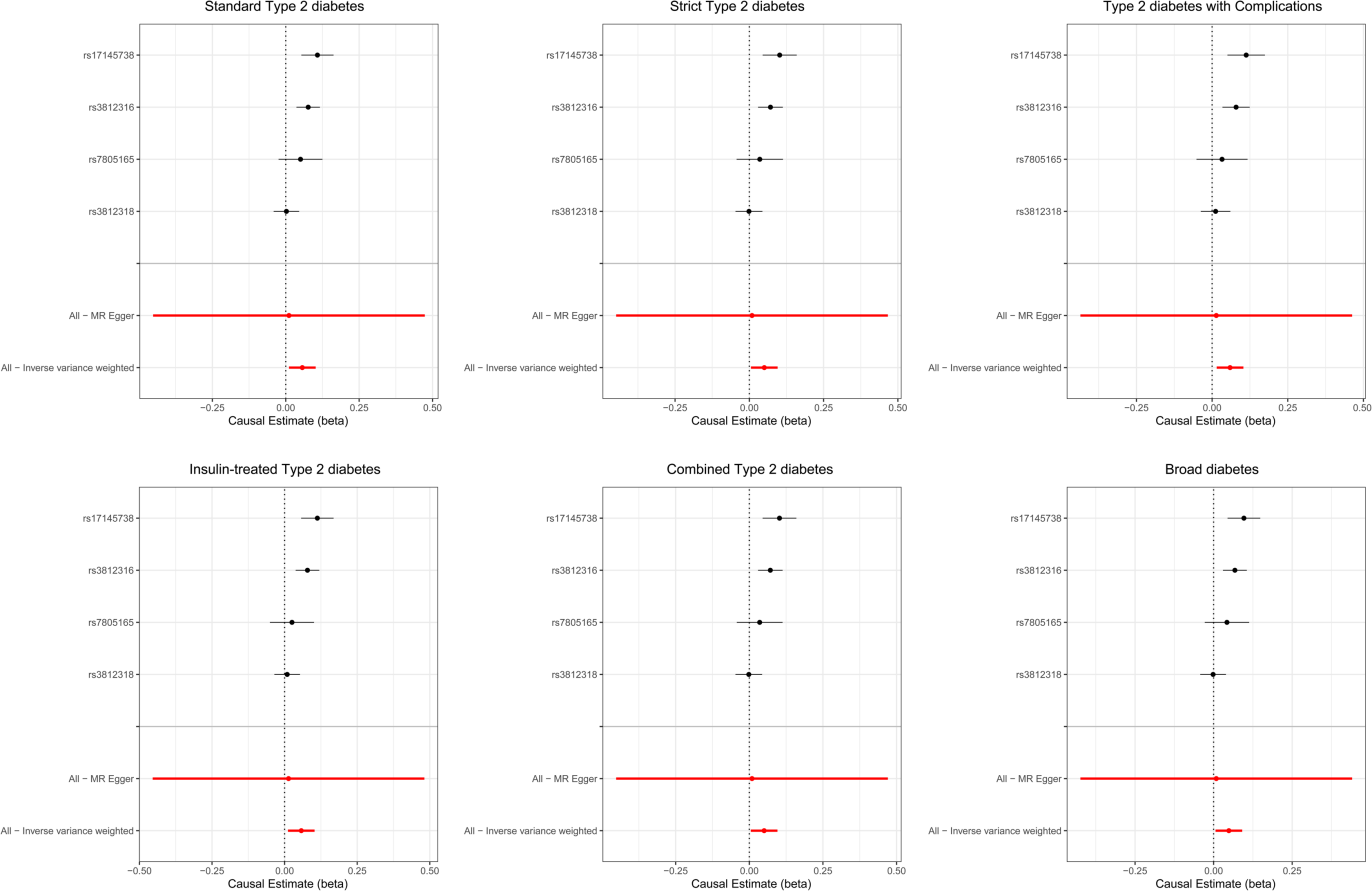
Forest plots showing the causal estimates of genetically predicted *MLXIPL* expression on Type 2 Diabetes across six phenotype definitions. Analyses were based on four cis-eQTL instruments, all with F-statistics > 10. Additional heterogeneity and pleiotropy statistics are reported in the main text and **Supplementary Tables 4**, **5**.

Visual inspection of scatter plots indicated consistent effect directions for genetically predicted *MLXIPL* expression across all six T2D phenotype definitions in FinnGen (**Figure 1**). In the primary analysis using the random-effects inverse-variance weighted (IVW) method, genetically predicted higher *MLXIPL* expression was associated with T2D (**Figure 2**; **Table 2**). Specifically, for the standard definition of T2D, the odds ratio (OR) was 1.06 (95% CI 1.01–1.11; P = 0.015). Similar estimates were observed across alternative clinical definitions, including strict T2D (OR = 1.05, P = 0.028), insulin-treated T2D (OR = 1.06, P = 0.014), and T2D with complications (OR = 1.06, P = 0.008).

**Table 2:** Two-sample Mendelian randomization estimates for genetically predicted *MLXIPL* expression on type 2 diabetes and renal damage (UACR).

Outcome	Method	nSNP	OR (95% CI)	P value
Standard type 2 diabetes	Inverse variance weighted	4	1.058 (1.011 to 1.107)	0.015
	Weighted median	4	1.066 (1.031 to 1.102)	<0.001
	MR Egger	4	1.011 (0.636 to 1.606)	0.967
	Weighted mode	4	1.082 (1.031 to 1.135)	0.049
	Simple mode	4	1.076 (1.013 to 1.142)	0.097
Strict type 2 diabetes	Inverse variance weighted	4	1.052 (1.006 to 1.100)	0.028
	Weighted median	4	1.056 (1.021 to 1.092)	0.002
	MR Egger	4	1.009 (0.639 to 1.594)	0.973
	Weighted mode	4	1.075 (1.023 to 1.131)	0.066
	Simple mode	4	1.064 (0.994 to 1.139)	0.174
Type 2 diabetes with complications	Inverse variance weighted	4	1.061 (1.015 to 1.109)	0.008
	Weighted median	4	1.059 (1.021 to 1.098)	0.002
	MR Egger	4	1.014 (0.647 to 1.588)	0.958
	Weighted mode	4	1.082 (1.025 to 1.143)	0.065
	Simple mode	4	1.035 (0.958 to 1.118)	0.450
Insulin-treated type 2 diabetes	Inverse variance weighted	4	1.059 (1.012 to 1.109)	0.014
	Weighted median	4	1.055 (1.020 to 1.090)	0.002
	MR Egger	4	1.014 (0.635 to 1.618)	0.960
	Weighted mode	4	1.083 (1.029 to 1.139)	0.054
	Simple mode	4	1.028 (0.949 to 1.114)	0.548
Combined type 2 diabetes	Inverse variance weighted	4	1.052 (1.005 to 1.100)	0.029
	Weighted median	4	1.056 (1.019 to 1.094)	0.003
	MR Egger	4	1.009 (0.636 to 1.601)	0.973
	Weighted mode	4	1.075 (1.026 to 1.127)	0.057
	Simple mode	4	1.065 (0.990 to 1.145)	0.190
Broad diabetes	Inverse variance weighted	4	1.050 (1.006 to 1.095)	0.025
	Weighted median	4	1.057 (1.024 to 1.092)	0.001
	MR Egger	4	1.008 (0.654 to 1.554)	0.973
	Weighted mode	4	1.072 (1.025 to 1.122)	0.057
	Simple mode	4	1.067 (1.007 to 1.130)	0.117
			Beta (95% CI)	
UACR (overall population)	Inverse variance weighted	2	0.032 (0.007 to 0.057)	0.013
UACR (non-diabetic population)	Inverse variance weighted	2	0.025 (0.000 to 0.051)	0.049

MR, Mendelian randomization; T2D, type 2 diabetes; UACR, urinary albumin-to-creatinine ratio; OR, odds ratio; CI, confidence interval; IVW, inverse-variance weighted; SNP, single-nucleotide polymorphism.

In sensitivity analyses, the weighted median estimator yielded directionally consistent and statistically stronger associations compared to the IVW method across most endpoints (e.g., standard T2D: OR = 1.07, 95% CI 1.03–1.10; P < 0.001). Regarding instrument heterogeneity, Cochran’s Q statistics were significant for several outcomes (P_heterogeneity_ < 0.05; **Supplementary Table 4**). MR-Egger intercept tests yielded P values > 0.05 (all P_intercept_ > 0.05; **Supplementary Table 5**). Leave-one-out analyses showed that the exclusion of individual SNPs did not substantially alter the overall associations (**Supplementary Figure 1**).

### 3.3 Genetically predicted MLXIPL expression and UACR

**Figure 3:**
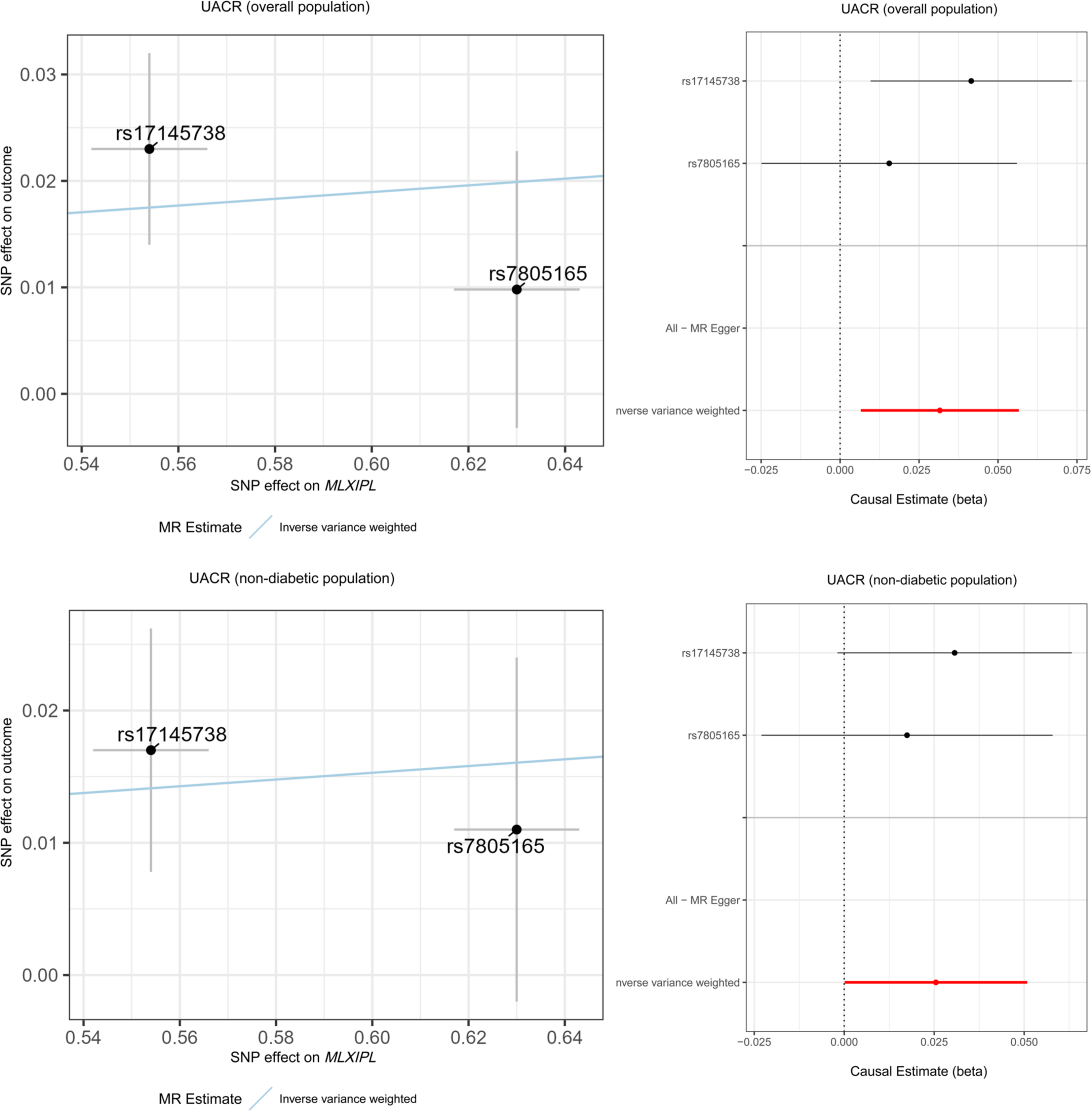
Mendelian randomization analysis of genetically predicted *MLXIPL* expression and urinary albumin-to-creatinine ratio (UACR) in the overall population and in the non-diabetic subset. Black points represent the retained SNP instruments and are labeled by rsID. UACR analyses were based on two retained SNP instruments after harmonization with the CKDGen datasets. Because only two variants were available, sensitivity methods requiring ≥3 SNPs were not applicable; heterogeneity statistics are provided in **Supplementary Table 4**.

We assessed the urinary albumin-to-creatinine ratio (UACR) using data from the CKDGen Consortium. In the discovery analysis involving the overall population (*N* = 54,450), IVW estimates showed that genetically predicted higher *MLXIPL* expression was associated with increased UACR (beta = 0.032, 95% CI 0.007–0.057; P = 0.013) (**Figure 3**; **Table 2**). In the non-diabetic stratification (*N* = 44,111), the association between genetically predicted *MLXIPL* expression and UACR was also statistically significant (beta = 0.025, 95% CI 0.0004–0.051; P = 0.049) (**Figure 3**; **Table 2**). Sensitivity analyses requiring ≥3 variants (e.g., MR-Egger) were not performed due to the limited number of instruments (nSNP = 2). Cochran’s Q statistic showed no evidence of heterogeneity in either the overall population (P_heterogeneity_ = 0.323) or the non-diabetic dataset (P_heterogeneity_ = 0.618) (**Supplementary Table 4**).

### 3.4 Transcriptomic characterization and intra-DKD stratification

**Figure 4:**
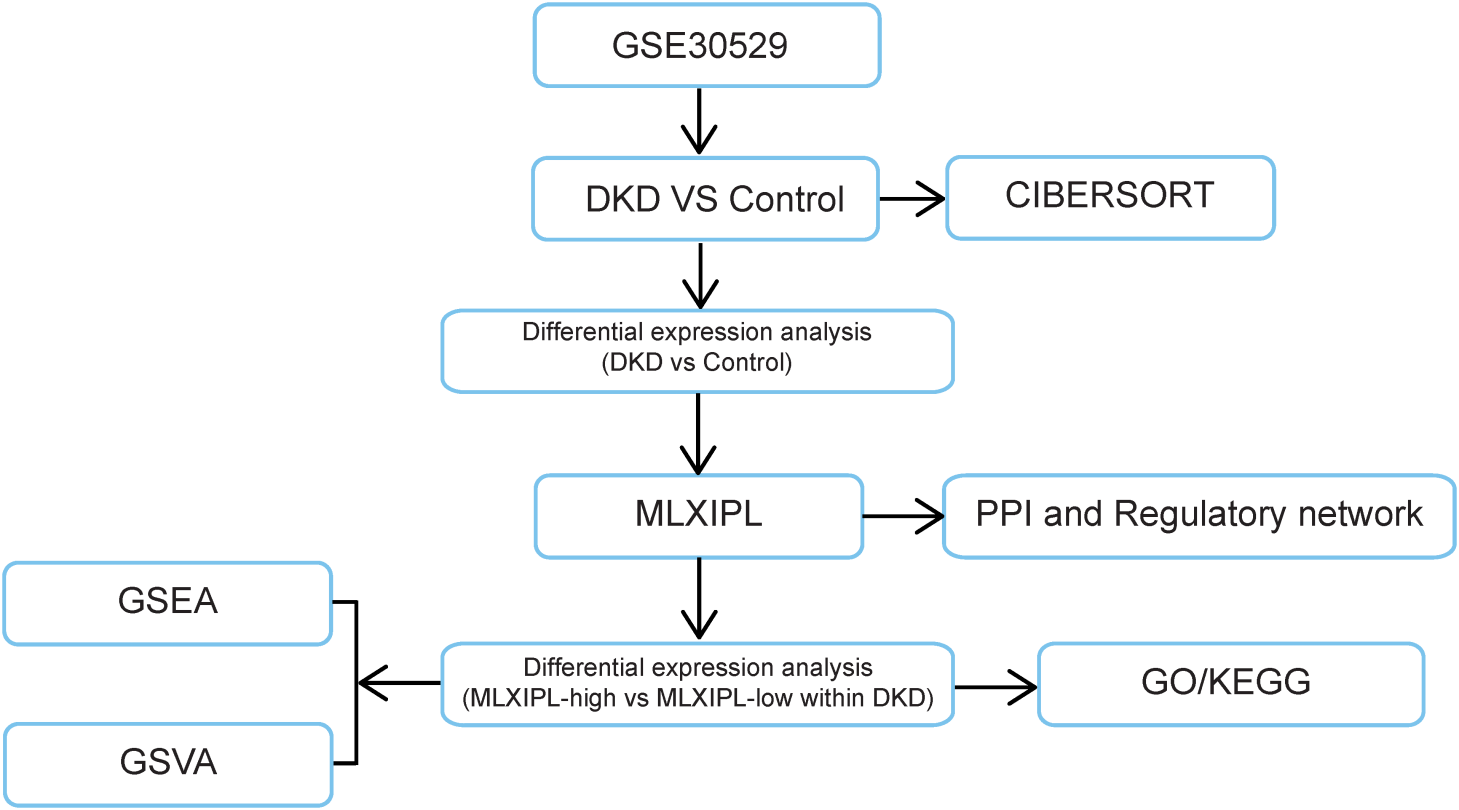
Overview of the integrative transcriptomic and bioinformatics analytical workflow.

**Figure 5:**
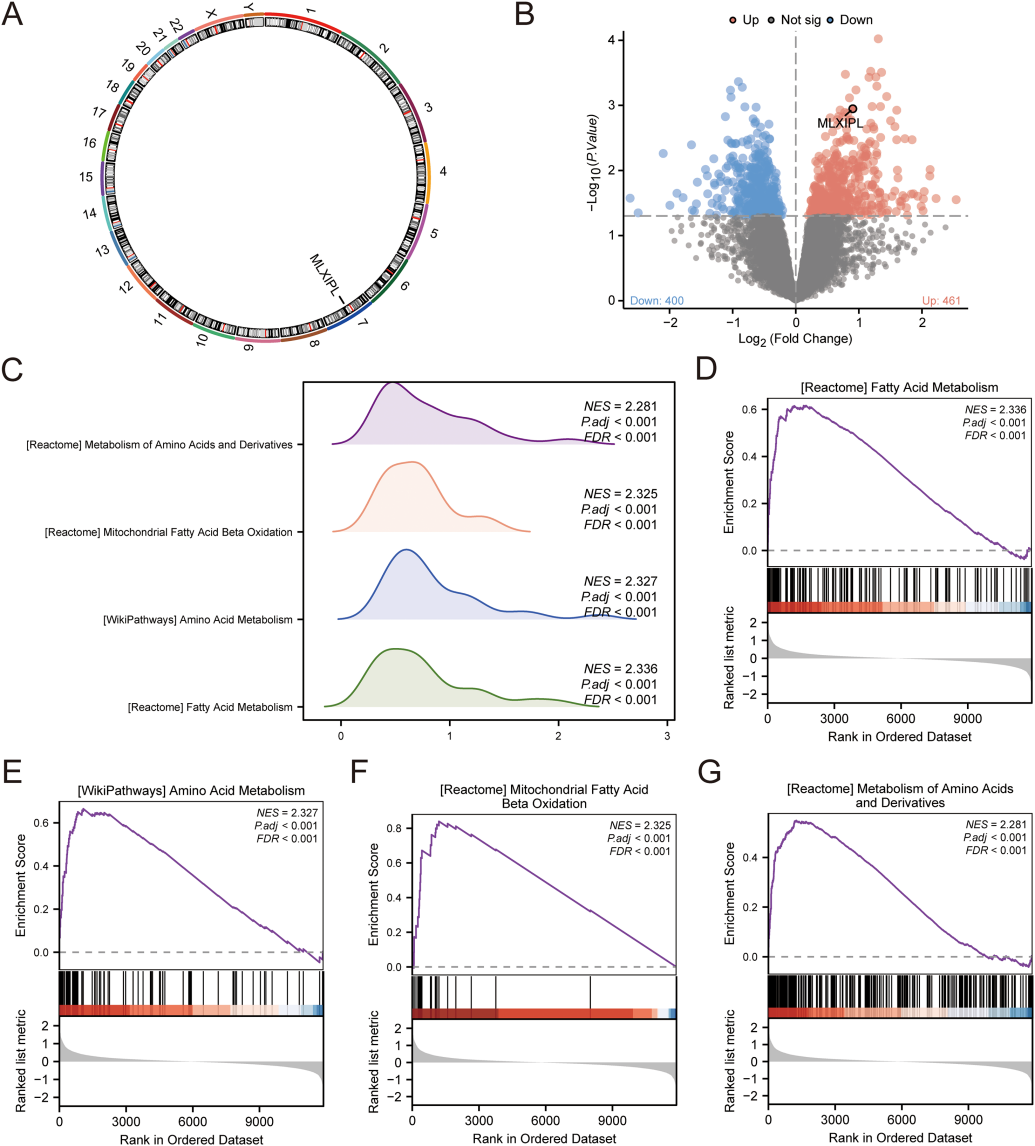
Transcriptomic analysis and GSEA of *MLXIPL*-associated pathways in diabetic kidney diesase. **(A)** Chromosomal location of the *MLXIPL* gene on chromosome 7. **(B)** Volcano plot showing differentially expressed genes from the *MLXIPL*-high versus *MLXIPL*-low comparison in GSE30529. DKD samples were stratified into *MLXIPL*-high and *MLXIPL*-low groups using the median *MLXIPL* expression as the cutoff (n = 5 per group).Genes were displayed using a nominal P value threshold of < 0.05 without an additional absolute fold-change cutoff; red and blue points indicate genes with positive and negative logFC values, respectively. **(C)** Ridge plot of the top four significantly enriched metabolic pathways in the *MLXIPL*-high group. **(D–G)** GSEA enrichment plots for Fatty acid metabolism **(D)**, Amino acid metabolism **(E)**, Mitochondrial fatty acid beta-oxidation **(F)**, and Metabolism of amino acids and derivatives **(G)**.

The overall bioinformatics and transcriptomic analysis workflow based on GSE30529 is summarized in **Figure 4**. We analyzed the transcriptomic profile of *MLXIPL* (chromosome 7; **Figure 5A**) using the GSE30529 dataset. In bulk-tissue comparisons, MLXIPL was significantly downregulated in DKD relative to controls (logFC = -0.72, P < 0.001). To further examine whether this apparent reduction was influenced by tissue composition and remodeling, we performed complementary sensitivity and sample-level analyses. In limma-based sensitivity models, adjustment for tubular integrity reduced the magnitude of differential expression, and separately adjusted models incorporating immune or fibrotic signatures further indicated that the bulk *MLXIPL* signal was sensitive to compositional features (**Supplementary Table 6**). Additional sample-level analyses showed that *MLXIPL* abundance correlated positively with tubular integrity (rho = 0.499, P = 0.019) and inversely with fibrotic (rho = -0.606, P = 0.003) and immune (rho = -0.623, P = 0.002) signatures (**Supplementary Figure 2**; **Supplementary Table 7**). In multivariable linear regression models, sequential adjustment for these compositional features attenuated, but did not abolish, the negative association between DKD status and *MLXIPL* expression (**Supplementary Table 8**).

In CIBERSORT analysis, immune cell fractions differed between DKD and control samples but showed no significant variation between the *MLXIPL*-high and *MLXIPL*-low subgroups within the DKD cohort (**Supplementary Figure 3**). We therefore retained the within-DKD median-based stratification strategy to investigate *MLXIPL*-associated transcriptomic programs while minimizing confounding from broad case–control compositional differences.

Differential expression analysis identified 951 genes at the nominal threshold (P < 0.05), with 461 upregulated and 490 downregulated transcripts in the *MLXIPL*-high subgroup (**Figure 5B**). In Gene Set Enrichment Analysis (GSEA), the *MLXIPL*-high subgroup exhibited significant enrichment of Fatty Acid Metabolism (NES = 2.34; **Figure 5D**), Amino Acid Metabolism (NES = 2.33; **Figure 5E**), and Mitochondrial Fatty Acid β-Oxidation (NES = 2.33; **Figure 5F**). These pathways were enriched with an FDR < 0.001 (**Supplementary Table 9**).

### 3.5 Divergent pathway activity profiles identified by GSVA

**Figure 6:**
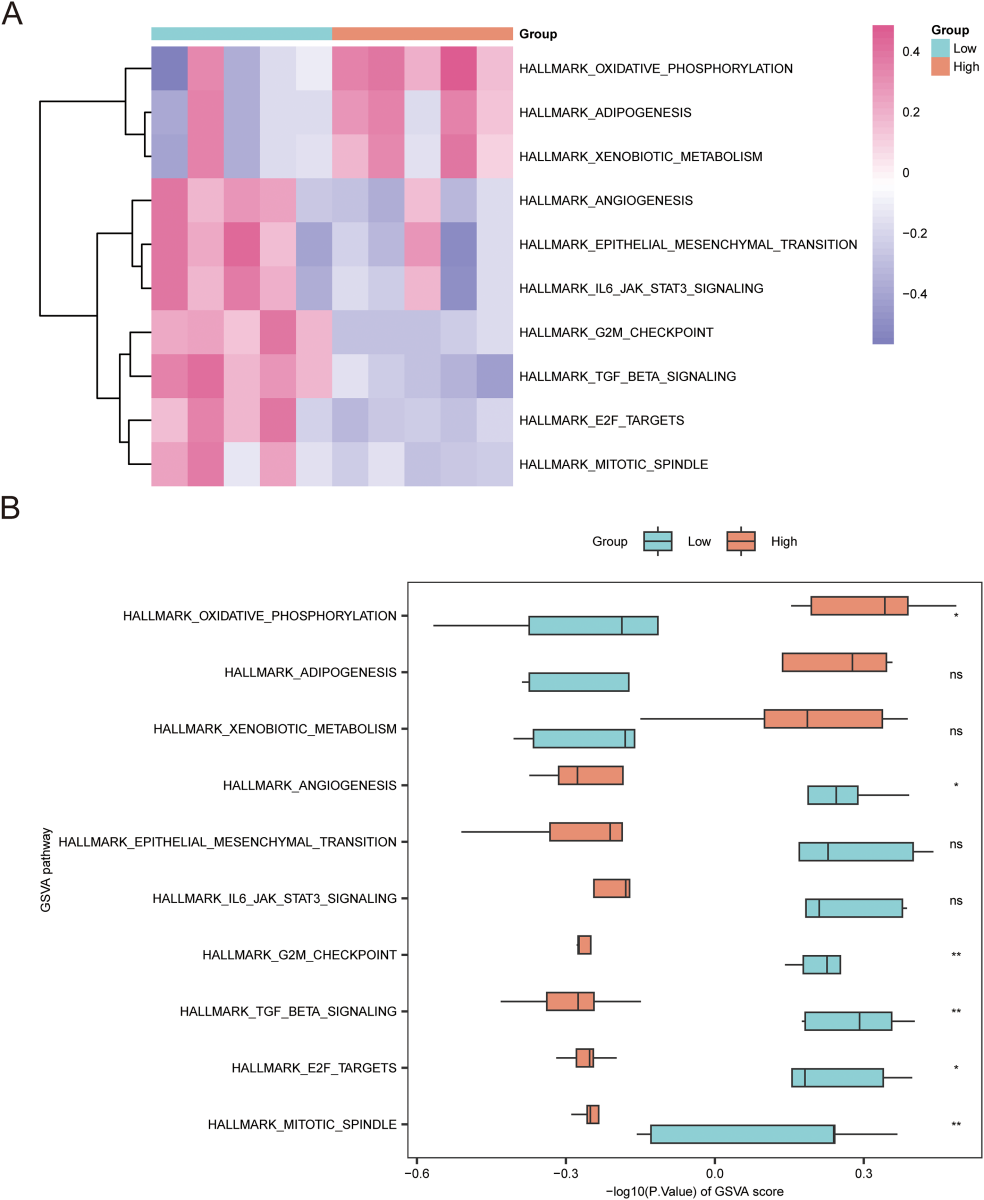
GSVA analysis of signaling pathways associated with *MLXIPL* expression. **(A)** Heatmap visualizing distinct pathway activity profiles between *MLXIPL*-high and *MLXIPL*-low groups based on the MSigDB Hallmark collection. DKD samples were stratified using the median *MLXIPL* expression as the cutoff (n = 5 per group). Red and blue represent high and low enrichment scores, respectively. **(B)** Box plots comparing GSVA scores for representative pathways between the *MLXIPL*-high and *MLXIPL*-low groups. Orange indicates the *MLXIPL*-high group; light blue indicates the *MLXIPL*-low group. Data are presented as median with interquartile range. *P < 0.05; **P < 0.01; ns, not significant.

To complement the ensemble-level GSEA and quantify pathway activity at single-sample resolution, we performed Gene Set Variation Analysis (GSVA) using the MSigDB Hallmark collection (**Supplementary Table 10**). Unsupervised clustering of GSVA enrichment scores revealed distinct signaling landscapes between the *MLXIPL*-high and *MLXIPL*-low DKD subgroups (**Figure 6A**).

In the *MLXIPL*-high subgroup, Oxidative Phosphorylation displayed significantly higher GSVA scores compared with the *MLXIPL*-low subgroup (**Figure 6B**). Adipogenesis showed a similar directional trend but did not reach statistical significance (**Figure 6B**). In contrast, the *MLXIPL*-low subgroup exhibited significantly higher activity scores for TGF β-signaling, epithelial–mesenchymal transition (EMT), and angiogenesis, together with proliferation-related programs including E2F Targets, G2M Checkpoint, and Mitotic Spindle (**Figure 6B**; all P < 0.05).

### 3.6 Functional enrichment analysis of representative *MLXIPL*-associated genes

**Figure 7:**
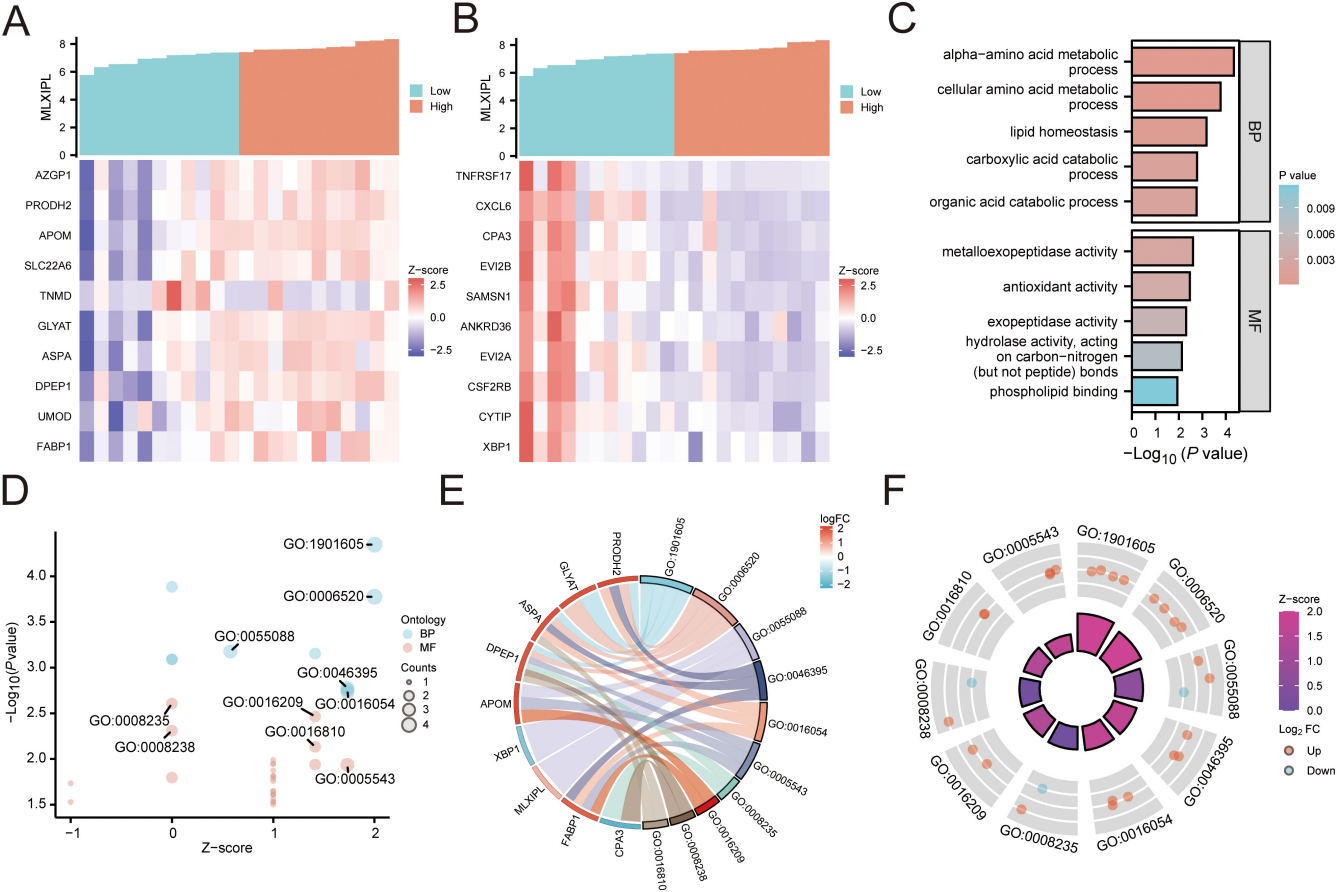
Functional enrichment analysis of genes associated with *MLXIPL* expression. **(A–B)** Heatmaps displaying representative differentially expressed genes derived from the *MLXIPL*-high versus *MLXIPL*-low comparison. **(A)** shows genes upregulated in the *MLXIPL*-high direction, and **(B)** shows genes upregulated in the *MLXIPL*-low direction. **(C–F)** Exploratory functional enrichment visualizations based on a representative gene set consisting of *MLXIPL* together with the top 10 positively associated and top 10 negatively associated genes selected from the *MLXIPL*-high versus *MLXIPL*-low comparison. **(C)** Bar plot ranking the top enriched Gene Ontology (GO) terms for Biological Process (BP) and Molecular Function (MF). **(D)** Bubble plot visualizing the significance and z-scores of representative GO terms. **(E)** Chord diagram illustrating the linkages between specific genes and metabolic GO terms. **(F)** Circos plot displaying gene-level log2 fold-changes (dots) and the overall z-score (inner ring) of the enriched terms.

We analyzed representative differentially expressed genes associated with *MLXIPL* from the *MLXIPL*-high versus *MLXIPL*-low comparison. In the *MLXIPL*-high direction, highly associated genes included *SLC22A6*, *FABP1*, *PRODH2*, and *UMOD* (**Figure 7A**). In the opposite direction, representative negatively associated genes included *TNFRSF17, CXCL6*, and *CSF2RB* (**Figure 7B**).

**Figures 7C–F** were generated from an exploratory functional enrichment analysis based on a representative gene set consisting of *MLXIPL* together with the top 10 positively associated and top 10 negatively associated genes from the *MLXIPL*-high versus *MLXIPL*-low comparison, rather than from all genes in the *MLXIPL*-high subgroup alone. Gene Ontology (GO) enrichment results for this gene set are summarized in **Supplementary Table 11**. For Biological Process (BP), significant enrichment was observed for alpha-amino acid metabolic process (adjusted P = 0.023, q = 0.015) and cellular amino acid metabolic process (adjusted P = 0.028, q = 0.018). The term lipid homeostasis showed a q-value of 0.038 (adjusted P = 0.059) (**Figure 7C**). Catabolic terms, specifically carboxylic acid catabolic process and organic acid catabolic process, yielded adjusted P values of 0.094 (q = 0.060) (**Supplementary Table 11**).

For Molecular Function (MF), the top ranked terms included phospholipid binding, metalloexopeptidase activity, and antioxidant activity (**Figure 7C**). These terms showed adjusted P values of approximately 0.089 (q= 0.053) (**Supplementary Table 11**). The bubble plot visualizes these statistics, showing that amino acid metabolic processes involved the highest number of genes (**Figure 7D**). The chord diagram maps specific genes to these metabolic terms; notably, genes such as *PRODH2, GPT2, ALDH6A1*, and *AGXT2* were linked to multiple amino acid metabolic categories (**Figure 7E**). The circle plot integrates gene-level logFC information from the *MLXIPL*-high versus *MLXIPL*-low comparison; positive z-scores indicate that several enriched terms were predominantly driven by genes upregulated in the *MLXIPL*-high direction (**Figure 7F**).

### 3.7 Protein–protein interaction and functional association networks of *MLXIPL*

**Figure 8:**
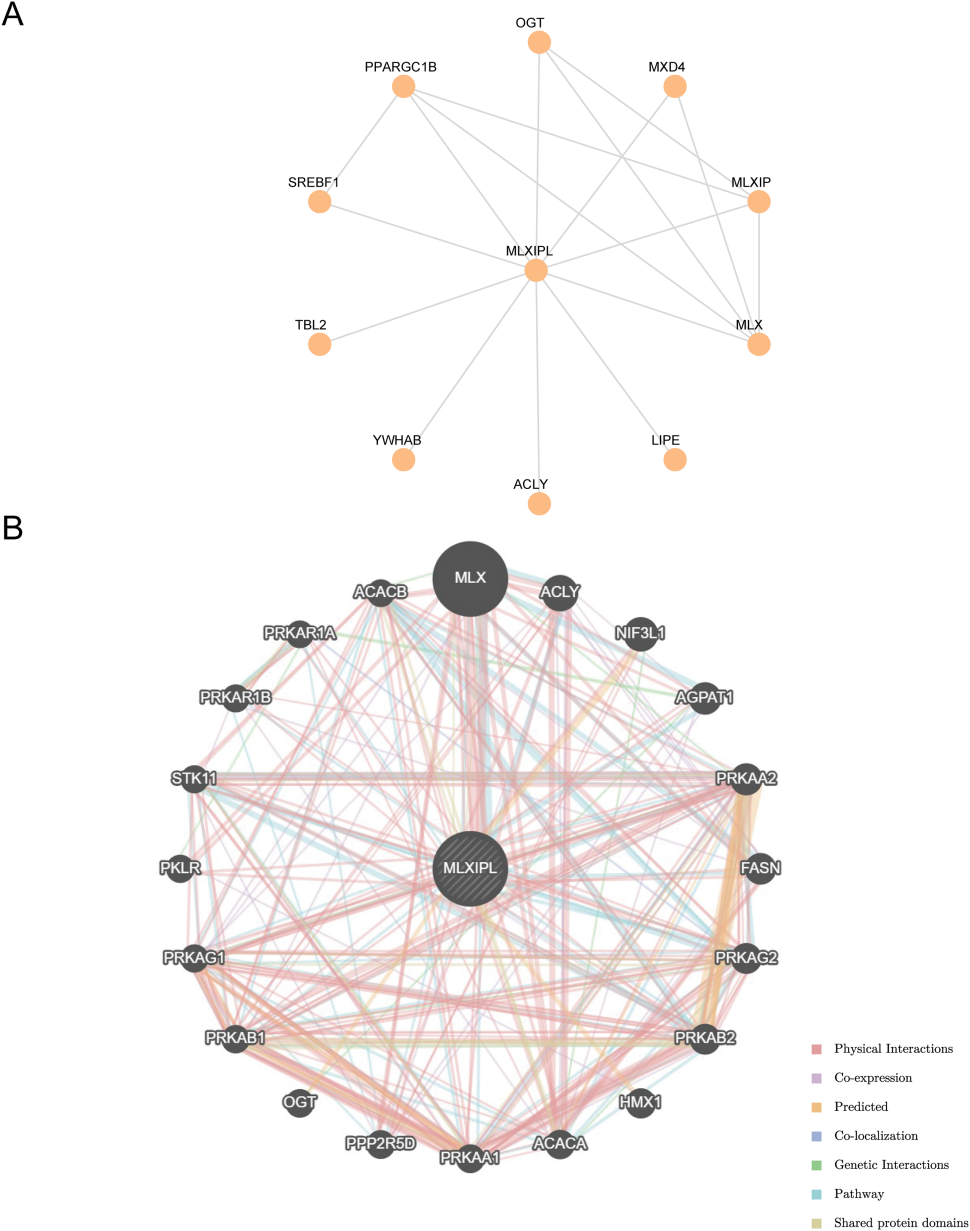
Protein–protein interaction and functional association networks of *MLXIPL*. **(A)** High-confidence protein–protein interaction (PPI) network constructed using the STRING database, displaying the top ten interaction partners of *MLXIPL*. **(B)** Functional association network generated by GeneMANIA. The diagram illustrates genes with shared functions or physical interactions with *MLXIPL*. Edge colors represent different lines of evidence (e.g., co-expression, physical interactions, and shared pathways).

We constructed a high-confidence protein–protein interaction (PPI) network using the STRING database with a minimum interaction score > 0.900 (**Figure 8A**). The top ten interaction partners identified were ACLY, LIPE, MLX, MLXIP, TBL2, PPARGC1B, SREBF1, YWHAB, OGT, and MXD4.

Functional associations were further analyzed using GeneMANIA, which integrates evidence from co-expression, physical interactions, co-localization, genetic interactions, predicted links, pathway co-membership, and shared protein domains (**Figure 8B**). In this network, *MLXIPL* exhibited connectivity with *ACACB, ACLY*, and *FASN* (**Figure 8B**).

### 3.8 Upstream regulatory landscape and chemical associations of *MLXIPL*

**Figure 9:**
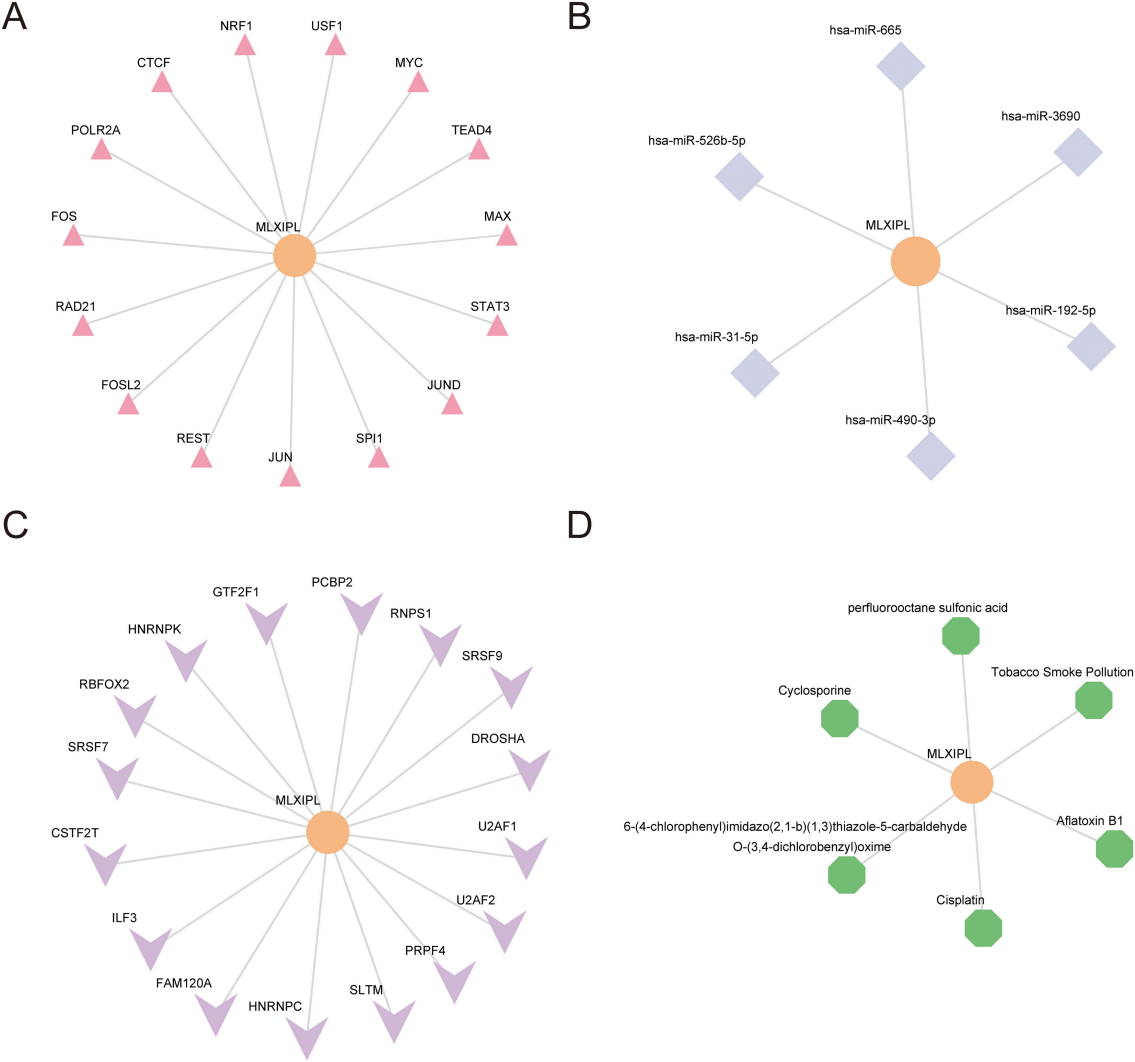
Upstream regulatory and chemical interaction landscapes of *MLXIPL*. **(A)** Transcription factor (TF)–gene interaction network. Pink triangles denote TFs predicted to regulate *MLXIPL*. **(B)** MicroRNA (miRNA)–gene interaction network. Light blue diamonds denote miRNAs targeting *MLXIPL*. **(C)** RNA-binding protein (RBP)–mRNA interaction network. Purple arrowheads denote RBPs binding to *MLXIPL* transcripts. **(D)** Chemical–gene interaction network. Green hexagons denote drugs or environmental chemicals associated with *MLXIPL*.

We integrated upstream transcriptional regulation, post-transcriptional interactions, and chemical–gene associations to visualize the regulatory network of *MLXIPL* using Cytoscape (**Figures 9A–D**).

ChIPBase annotation identified 15 transcription factors (TFs) with binding evidence at the *MLXIPL* locus (**Figure 9A**; **Supplementary Table 12**). The identified TFs included AP-1 family members (FOS, FOSL2, JUN, JUND), MYC/MAX complex components, and other regulators such as CTCF, NRF1, POLR2A, RAD21, REST, SPI1, STAT3, TEAD4, and USF1.

ENCORI analysis predicted six microRNAs targeting *MLXIPL*, forming the mRNA–miRNA interaction network (**Figure 9B**; **Supplementary Table 13**). These included hsa-miR-31-5p, hsa-miR-192-5p, hsa-miR-490-3p, hsa-miR-526b-5p, hsa-miR-665, and hsa-miR-3690.

StarBase analysis identified 16 RNA-binding proteins (RBPs) interacting with *MLXIPL* transcripts (**Figure 9C**; **Supplementary Table 14**). The network included splicing factors (e.g., U2AF1, U2AF2, SRSF7, SRSF9, RNPS1), heterogeneous nuclear ribonucleoproteins (HNRNPC, HNRNPK), and the microprocessor complex component DROSHA.

The Comparative Toxicogenomics Database (CTD) screen yielded six chemical or environmental agents associated with *MLXIPL* (**Figure 9D**; **Supplementary Table 15**). These included pharmaceutical agents (Cisplatin, Cyclosporine), environmental toxins (Aflatoxin B1, Tobacco Smoke Pollution), Perfluorooctane sulfonic acid, and the compound 6-(4-chlorophenyl) imidazo(2,1-b) (1,3) thiazole-5-carbaldehyde O-(3,4-dichlorobenzyl) oxime.

### 3.9 Expression of ChREBP/*Mlxipl* and metabolic targets in diabetic models

**Figure 10:**
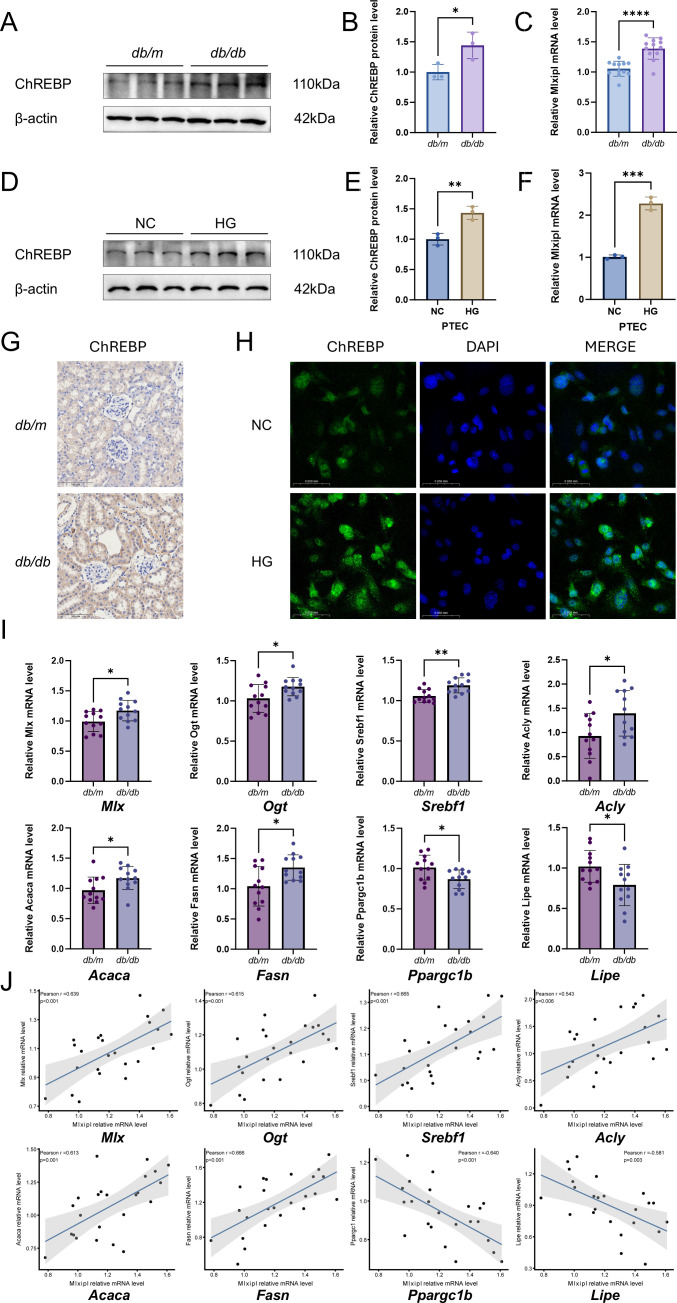
Experimental validation of *MLXIPL* upregulation and metabolic network consistency in diabetic models. **(A–C)** Assessment of ChREBP expression in kidney tissues from *db/m* and *db/db* mice. Representative Western blots **(A)** and densitometric quantification **(B)** of ChREBP protein levels. For animal Western blot analyses, densitometric quantification was performed using all 12 mice per group. Full-length immunoblot images are provided in **Supplementary Figure 4**. **(C)** Relative mRNA expression of *Mlxipl*. **(D–F)** Assessment of ChREBP expression in primary proximal tubular epithelial cells (PTECs) under normal glucose (NC) and high glucose (HG) conditions. Representative Western blots **(D)**, protein quantification **(E)**, and mRNA levels **(F)**. **(G)** Representative immunohistochemistry (IHC) staining of ChREBP in mouse kidney sections. Scale bar: 100 μm. **(H)** Representative immunofluorescence (IF) staining of ChREBP (green) in PTECs; nuclei were counterstained with DAPI (blue). Scale bar: 50 μm. **(I)** Relative mRNA expression of *MLXIPL*-interacting metabolic genes in mouse kidneys. **(J)** Pearson correlation analysis between *Mlxipl* expression and downstream metabolic targets in kidney tissues.Data are presented as mean ± SD. *P < 0.05, **P < 0.01, ***P < 0.001, ****P < 0.0001.

We examined ChREBP protein and *Mlxipl* mRNA levels in kidney tissues from *db/m* and *db/db* mice (**Figure 10**). Immunoblotting analyses indicated that ChREBP protein abundance was higher in *db/db* mice compared to *db/m* controls (**Figure 10A**), and densitometric quantification showed a significant increase (**Figure 10B**). *Mlxipl* mRNA levels were also significantly elevated in the *db/db* group (**Figure 10C**). In immunohistochemistry assays, ChREBP staining intensity was higher in diabetic kidneys compared to controls (**Figure 10G**).

In primary proximal tubular epithelial cells (PTECs), stimulation with high glucose (HG; 25 mM) for 48 h resulted in higher ChREBP protein levels compared to normal glucose conditions (**Figures 10D, E**). *Mlxipl* mRNA expression was also higher in the HG group (**Figure 10F**). Immunofluorescence staining showed increased ChREBP signal intensity in HG-treated cells (**Figure 10H**).

We further quantified the expression of *Mlxipl*-related metabolic genes in mouse kidney tissues. Compared to *db/m* controls, *db/db* mice exhibited higher expression levels of *Mlx, Ogt, Srebf1, Acly, Acaca*, and *Fasn*, and lower expression levels of *Ppargc1b* and *Lipe* (**Figure 10I**). In Pearson correlation analyses across the mouse kidney samples (*n* = 24), *Mlxipl* expression correlated positively with *Mlx* (r = 0.639, P < 0.001), *Ogt* (r = 0.615, P = 0.001), *Srebf1* (r = 0.665, P < 0.001), *Acly* (r = 0.543, P = 0.006), *Acaca* (r = 0.613, P = 0.001), and *Fasn* (r = 0.666, P < 0.001). Negative correlations were observed with *Ppargc1b* (r = -0.640, P < 0.001) and *Lipe* (r = -0.581, P = 0.003) (**Figure 10J**).

## 4 Discussion

Diabetic kidney disease (DKD) represents a chronic convergence of systemic nutrient overload and local maladaptive remodeling, culminating in progressive fibrosis and renal functional decline (1, 6). A major obstacle to mechanism-informed therapy is identifying the transcriptional regulators that couple systemic metabolic excess to kidney-intrinsic injury programs (10). In this study, by integrating two-sample Mendelian randomization (MR), kidney transcriptomic stratification, network biology, and *in vivo*/*in vitro* validation, we provide convergent evidence that ChREBP (encoded by *MLXIPL*) constitutes a pathogenic metabolic node linking diabetes susceptibility to renal damage. Our findings support *MLXIPL*/ChREBP-associated maladaptive metabolic remodeling in DKD, while also indicating that bulk-tissue *MLXIPL* abundance in advanced human disease is strongly shaped by tissue composition and structural remodeling.

A cornerstone of this study is the genetic support for causality. Using independent GWAS resources, genetically predicted higher *MLXIPL* expression was consistently associated with increased risk of type 2 diabetes (T2D) across multiple clinical phenotype definitions (30). Extending beyond glycemic outcomes, our analysis revealed that genetically predicted *MLXIPL* expression was also associated with elevated UACR, a causal effect that persisted in a non-diabetic stratification (26). This finding is clinically significant, as it challenges the traditional view that ChREBP-mediated renal injury is solely a secondary consequence of systemic hyperglycemia. Instead, it suggests a kidney-intrinsic vulnerability, where genetically driven hyperactivity of the ChREBP axis may sensitize the renal parenchyma to injury even in the absence of overt diabetes. This aligns with emerging concepts of “metabolic memory” and genetic susceptibility in CKD progression, positioning *MLXIPL* not merely as a passive responder to glucose, but as an upstream effector of renal risk. Importantly, these genetic findings reflect disease susceptibility rather than the average transcript abundance observed in advanced bulk kidney tissue and therefore are not expected to map directly onto late-stage human transcriptomic readouts. This interpretation is also concordant with prior loss-of-function studies suggesting that ChREBP contributes to diabetic renal injury (17, 18). In this context, our study extends the existing literature by linking *MLXIPL*/ChREBP to human genetic susceptibility, renal damage phenotypes, and kidney-wide transcriptomic programs, thereby strengthening the translational relevance of this pathway.

However, reconciling genetic susceptibility with the lower bulk-tissue expression of *MLXIPL* observed in human DKD requires careful interpretation. Our additional analyses suggest that reduced bulk-tissue *MLXIPL* abundance should not be interpreted in a simplistic manner as evidence against pathogenic relevance. Instead, *MLXIPL* abundance correlated positively with tubular integrity and inversely with fibrotic and immune signatures, indicating that its tissue-level signal is strongly influenced by structural and compositional changes in advanced disease. Notably, adjustment for tubular integrity and fibro-inflammatory signatures attenuated, but did not fully eliminate, the negative association with DKD. Thus, compositional shift appears to be an important—but not exclusive—contributor to the apparent reduction of *MLXIPL* in bulk human DKD tissue. In this context, *MLXIPL*/ChREBP may be better interpreted as a tubule-enriched nutrient-sensing regulator whose apparent bulk abundance becomes attenuated as DKD progresses and metabolically active tubular compartments are lost or replaced by fibro-inflammatory components. This interpretation is consistent with recent single-cell atlases of the diabetic kidney, which show that proximal tubules undergo marked metabolic and phenotypic remodeling during progression to fibrosis (48).

From an integrative perspective, our multi-omics and experimental data are consistent with a state of maladaptive lipogenic–oxidative imbalance. ChREBP is canonically known to couple glycolysis to *de novo* lipogenesis (11, 12). In our diabetic models, we observed coordinated upregulation of *MLXIPL* and downstream lipogenic targets including *Acly, Acaca*, and *Fasn*. At the same time, this lipogenic pattern was accompanied by inverse associations with *Ppargc1b* and *Lipe*, suggesting that anabolic drive may not be matched by proportional support for oxidative or lipid-handling programs. Although these data do not directly establish renal lipotoxicity, they are consistent with a scenario in which nutrient sensing through ChREBP contributes to maladaptive metabolic remodeling under diabetic stress. Our GSVA results further associated the *MLXIPL*-low subgroup with profibrotic, angiogenic, and proliferative programs, supporting the view that transcriptomic differences around *MLXIPL* reflect distinct pathological states within DKD rather than a simple binary of “high equals harmful” and “low equals protective”.

From a translational perspective, these findings suggest that *MLXIPL*/ChREBP represents a biologically and mechanistically relevant axis connecting nutrient excess to maladaptive renal metabolic rewiring. These observations are also relevant in the context of sodium-glucose cotransporter 2 inhibitors (SGLT2i), which have established renoprotective effects in DKD and broader CKD. Preclinical studies suggest that SGLT2 inhibition may attenuate renal lipid accumulation together with suppression of ChREBP-linked lipogenic programs; however, whether the kidney-protective effects of SGLT2i directly converge on *MLXIPL*/ChREBP signaling remains to be clarified (22, 23). The regulatory networks constructed herein, placing *MLXIPL* within a dense cluster of metabolic regulators and upstream stress responders, offer a rich resource for hypothesis generation. Therapeutic strategies may therefore need to restrain pathological *MLXIPL*/ChREBP-driven outputs—particularly the lipogenic arm—while preserving baseline metabolic functions essential for tubular physiology. Emerging metabolic modulators that uncouple lipogenesis from glucose handling offer a promising template for such precision interventions (49, 50). Given the potential kidney-intrinsic effects identified by our MR analysis, renal-targeted modulation of ChREBP-dependent lipogenic programs may represent a potential strategy to halt the progression from metabolic stress to fibrosis.

Several limitations of this study warrant consideration. While MR infers causality, it does not spatially resolve the tissue of action; however, our consistent results in kidney cells (*in vitro*) and tissue (*in vivo*) help bridge this gap. As an additional tissue-relevance check, we cross-referenced the lead blood cis-eQTL instruments in GTEx v10 Kidney Cortex eQTL data. Two of the four primary SNPs (rs17145738 and rs3812316) were identifiable and showed directionally consistent positive effects on *MLXIPL* expression, although neither reached nominal significance. These findings provide limited directional support for concordance between blood and kidney cortex regulation, but the small sample size of kidney cortex eQTL resources, limited overlap of variants, and variant-level representation constraints precluded a sufficiently powered kidney-specific validation MR. In addition, significant Cochran’s Q values were observed for several T2D outcomes, indicating between-instrument heterogeneity. Although we used random-effects IVW as the primary estimator and observed directionally consistent results with the weighted median method, these findings suggest that the IVW estimates should be interpreted with caution. Transcriptomic inference was based on a relatively small public bulk kidney cohort (GSE30529; 10 DKD and 12 control samples); although this dataset is well-characterized and widely used in DKD transcriptomic studies, its limited sample size may reduce statistical power and constrain the generalizability of differential expression estimates. We therefore interpreted the transcriptomic findings in conjunction with within-DKD stratification, compositional analyses, MR evidence, and experimental validation. In addition, the apparent discrepancy between human bulk transcriptomic data and experimental models underscores the stage-, compartment-, and context-dependent nature of *MLXIPL*/ChREBP regulation in DKD. Single-cell or spatial transcriptomics are still needed to localize *MLXIPL*-driven programs to specific tubular subpopulations (19). The predicted regulatory links require further biochemical validation to dissect the precise molecular hierarchy.

In summary, by integrating genetic inference, kidney transcriptomics, network biology, and experimental validation, our study supports *MLXIPL*/ChREBP as a disease-relevant nutrient-sensing regulator linked to maladaptive metabolic remodeling and renal injury in DKD. Rather than serving as a uniformly elevated bulk transcriptomic marker, *MLXIPL*/ChREBP appears to be context-dependent, with its apparent abundance in human kidney tissue shaped by disease stage and tissue composition.

## Data Availability

The kidney transcriptomic dataset analyzed in this study is publicly available from the Gene Expression Omnibus (GEO) under accession GSE30529. Summary statistics used for Mendelian randomization are available from the eQTLGen Consortium (cis-eQTLs for MLXIPL), FinnGen (T2D-related phenotypes), and CKDGen/IEU OpenGWAS (UACR traits). The analysis scripts and processed data generated during the current study are available from the corresponding authors upon reasonable request.

## Supplementary material

Supplementary Figure 1 Leave-one-out sensitivity analysis for the causal effect of *MLXIPL* expression on Type 2 Diabetes.

Supplementary Figure 2 Immune cell infiltration analysis estimated by CIBERSORT. **(A–B)** Immune cell composition in Control and Diabetic Kidney Disease (DKD) groups. **(A)** Stacked bar chart displaying the relative fractions of 22 immune cell types. **(B)** Box plots displaying the infiltration abundance of immune cells in Control versus DKD samples. **(C–D)** Immune cell composition in *MLXIPL*-low and *MLXIPL*-high DKD subgroups. **(C)** Stacked bar chart of immune cell fractions. **(D)** Box plots displaying the infiltration abundance of immune cells in *MLXIPL*-low versus *MLXIPL*-high subgroups. P-values are indicated above the corresponding box plots. Data are presented as median with interquartile range. *P < 0.05, **P < 0.01, ***P < 0.001, ****P < 0.0001.

Supplementary Figure 3 Associations of bulk-tissue MLXIPL expression with tubular integrity, fibrosis, and immune signatures in GSE30529. **(A)** Correlation between MLXIPL expression and tubular integrity score in kidney tubulointerstitial samples from GSE30529. **(B)** Correlation between MLXIPL expression and fibrosis score. **(C)** Correlation between MLXIPL expression and immune signature score. **(D)** Residualized MLXIPL expression after adjustment for tubular integrity, fibrosis, and immune signatures, shown by group. Residual values were derived from a linear model including these compositional features. Each dot represents one sample. Correlation coefficients were calculated using Spearman’s rank correlation test.

Supplementary Figure 4 Technical replicates and full-length immunoblot images for ChREBP detection in diabetic models. **(A)** Technical replicate western blots of ChREBP in kidney tissues from db/m and db/db mice (Repeat 1–3). **(B)** Technical replicate western blots of ChREBP in primary proximal tubular epithelial cells (PTECs) (Repeat 1–3). **(C–D)** Full-length immunoblot images used for densitometric quantification of ChREBP in mouse kidney tissues, including db/m 1–6 and db/db 1–6 **(C)**, and db/m 7–12 and db/db 7–12 **(D)**. β-Actin was used as the loading control. The quantitative analysis shown in **Figure 10B** was based on all 12 mice per group.
