# Supplementary figures and images for: Integrated causal inference, kidney transcriptomics, and experimental validation identify ChREBP (MLXIPL) as a driver of maladaptive metabolic remodeling in diabetic kidney disease
Source: Front Endocrinol (Lausanne). 2026 Apr 15;17:1809567. doi: 10.3389/fendo.2026.1809567 (PMC13125001; doi:10.3389/fendo.2026.1809567)

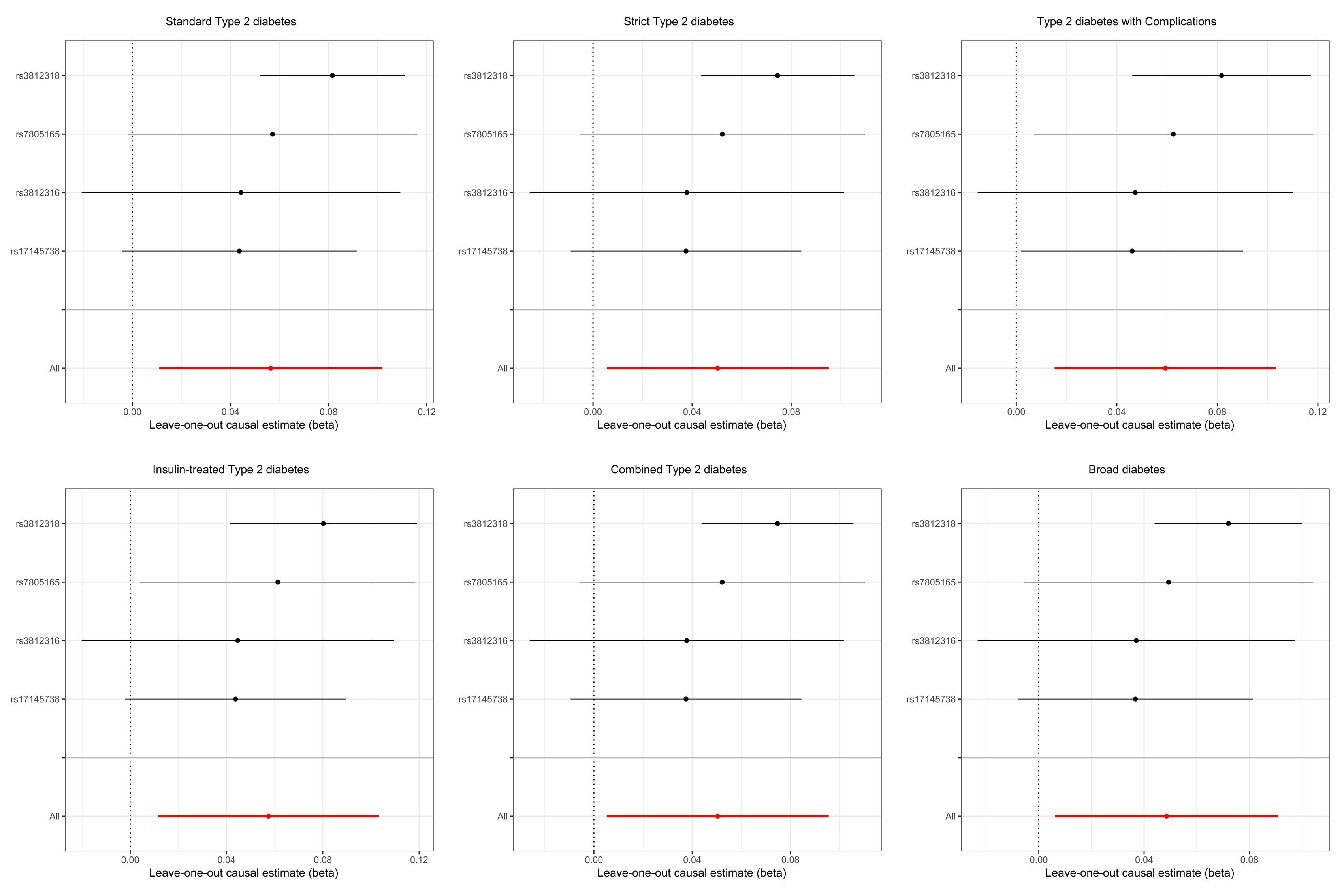

Supplement: Supplementary Figure 1 — Leave-one-out sensitivity analysis for the causal effect of MLXIPL expression on Type 2 Diabetes. [file Image1.tif]

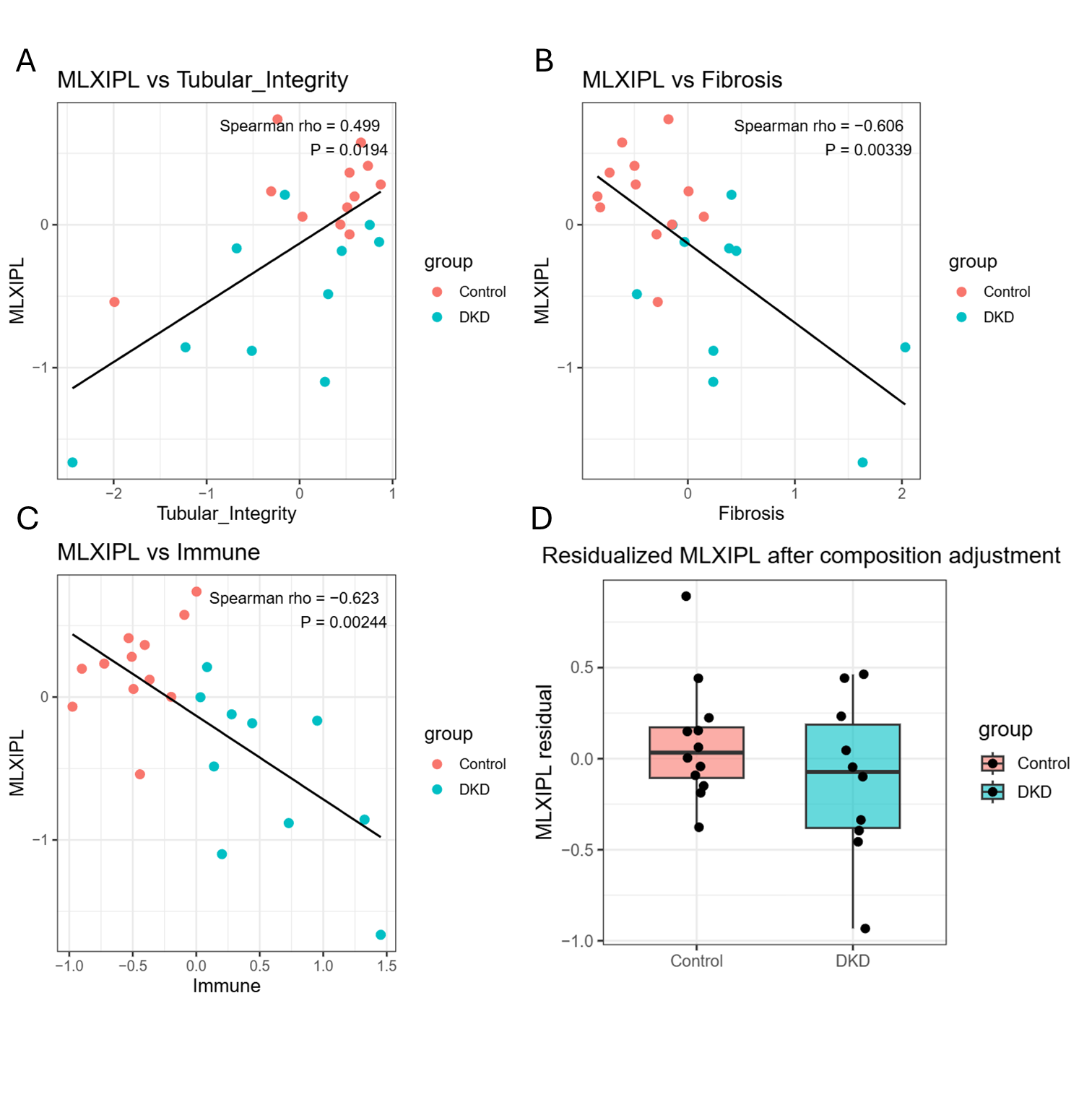

Supplement: Supplementary Figure 2 — Immune cell infiltration analysis estimated by CIBERSORT. (A–B) Immune cell composition in Control and Diabetic Kidney Disease (DKD) groups. (A) Stacked bar chart displaying the relative fractions of 22 immune cell types. (B) Box plots displaying the infiltration abundance of immune cells in Control versus DKD samples. (C–D) Immune cell composition in MLXIPL-low and MLXIPL-high DKD subgroups. (C) Stacked bar chart of immune cell fractions. (D) Box plots displaying the infiltration abundance of immune cells in MLXIPL-low versus MLXIPL-high subgroups. P-values are indicated above the corresponding box plots. Data are presented as median with interquartile range. *P < 0.05, **P < 0.01, ***P < 0.001, ****P < 0.0001. [file Image2.tif]

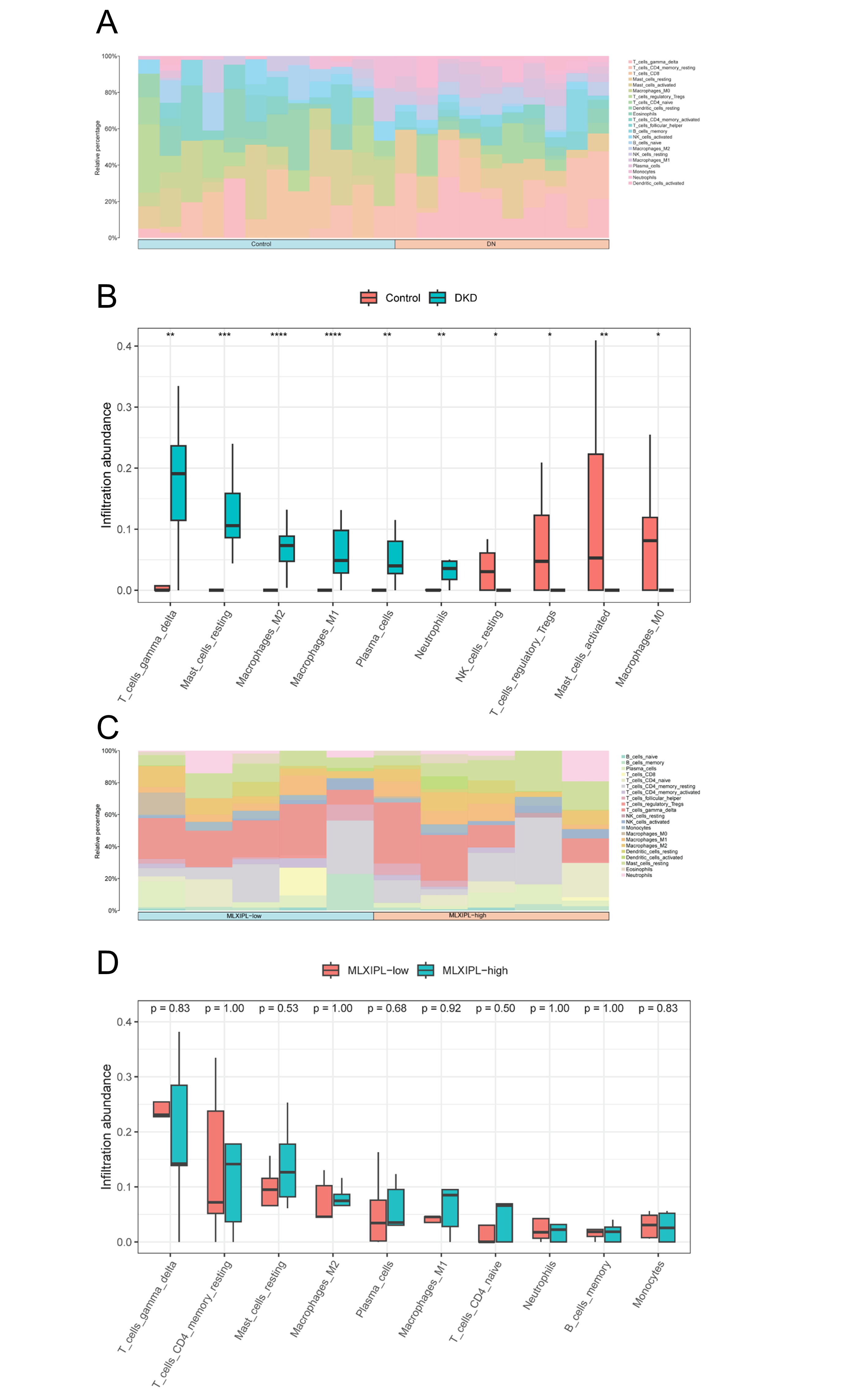

Supplement: Supplementary Figure 3 — Associations of bulk-tissue MLXIPL expression with tubular integrity, fibrosis, and immune signatures in GSE30529. (A) Correlation between MLXIPL expression and tubular integrity score in kidney tubulointerstitial samples from GSE30529. (B) Correlation between MLXIPL expression and fibrosis score. (C) Correlation between MLXIPL expression and immune signature score. (D) Residualized MLXIPL expression after adjustment for tubular integrity, fibrosis, and immune signatures, shown by group. Residual values were derived from a linear model including these compositional features. Each dot represents one sample. Correlation coefficients were calculated using Spearman’s rank correlation test. [file Image3.tif]

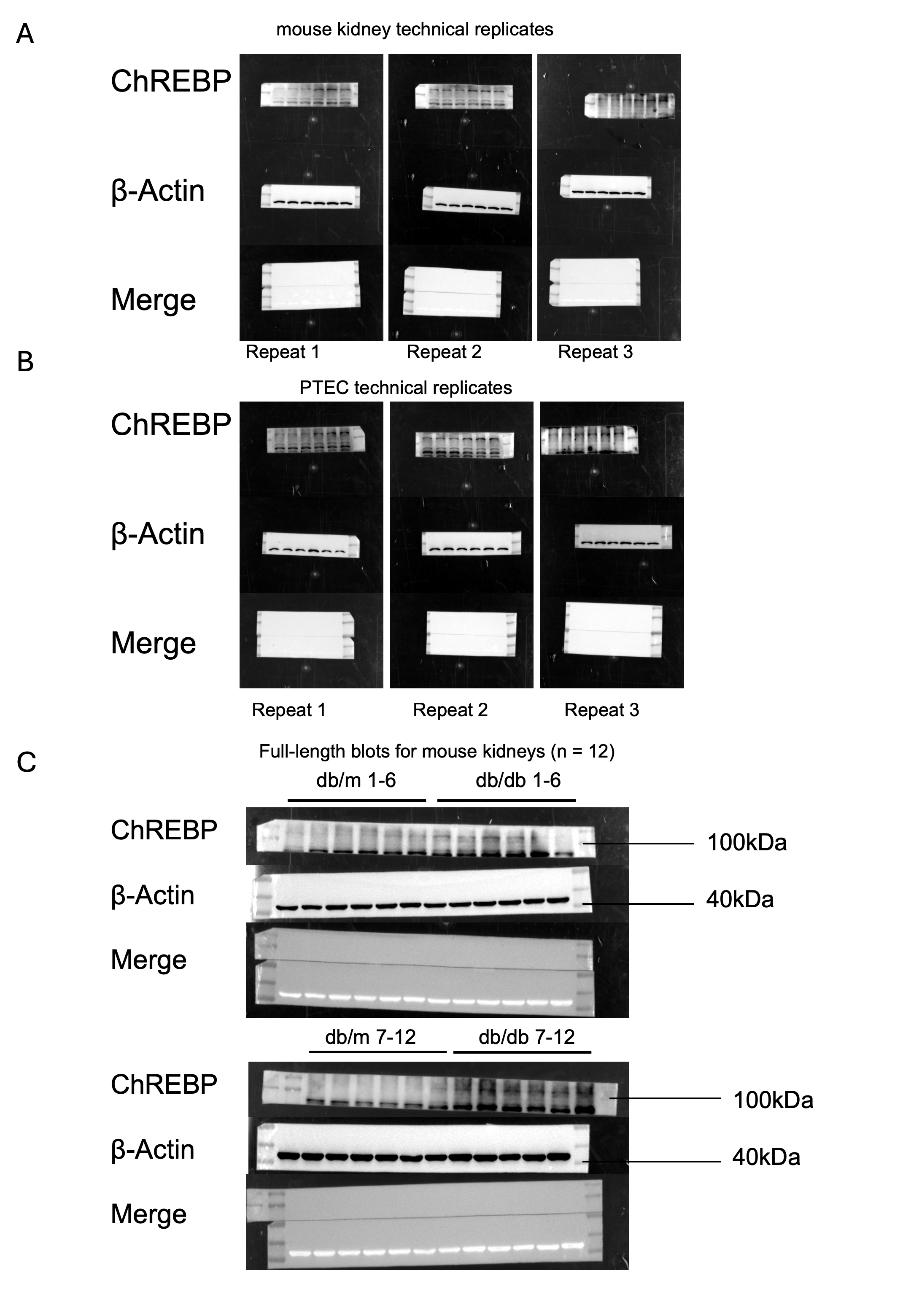

Supplement: Supplementary Figure 4 — Technical replicates and full-length immunoblot images for ChREBP detection in diabetic models. (A) Technical replicate western blots of ChREBP in kidney tissues from db/m and db/db mice (Repeat 1–3). (B) Technical replicate western blots of ChREBP in primary proximal tubular epithelial cells (PTECs) (Repeat 1–3). (C–D) Full-length immunoblot images used for densitometric quantification of ChREBP in mouse kidney tissues, including db/m 1–6 and db/db 1–6 (C), and db/m 7–12 and db/db 7–12 (D). β-Actin was used as the loading control. The quantitative analysis shown in Figure 10B was based on all 12 mice per group. [file Image4.tiff]
