# Supplementary material for: Integrated causal inference, kidney transcriptomics, and experimental validation identify ChREBP (MLXIPL) as a driver of maladaptive metabolic remodeling in diabetic kidney disease
Source: Front Endocrinol (Lausanne). 2026 Apr 15;17:1809567. doi: 10.3389/fendo.2026.1809567 (PMC13125001; doi:10.3389/fendo.2026.1809567)
Supplement: Supplementary file 5 [file Table1.docx]

### Table S1 GEO Microarray Chip Information

|  | GSE30529 |
| --- | --- |
| Platform | GPL571 |
| Species | Homo sapiens |
| Tissue | Kidney |
| Samples in DKD group | 10 |
| Samples in Control group | 12 |
| Reference | 21752957 |

GEO, Gene Expression Omnibus; DKD,Diabetic kidney disease.
