# Supplementary material for: Integrated causal inference, kidney transcriptomics, and experimental validation identify ChREBP (MLXIPL) as a driver of maladaptive metabolic remodeling in diabetic kidney disease
Source: Front Endocrinol (Lausanne). 2026 Apr 15;17:1809567. doi: 10.3389/fendo.2026.1809567 (PMC13125001; doi:10.3389/fendo.2026.1809567)
Supplement: Supplementary file 6 [file Table2.docx]

**TableS2.primer sequences used for mouse qRT–PCR**

| **Gene symbol** | **Primer** | **Sequence (5’→3’)** |  |
| --- | --- | --- | --- |
| **Mlxipl** | Forward | GAGTGCTTGAGCCTGGCTTACA |  |
|  | Reverse | GCTCTCCAGATGGCGTTGTTCA |  |
| **Srebf1** | Forward | CGACTACATCCGCTTCTTGCAG |  |
|  | Reverse | CCTCCATAGACACATCTGTGCC |  |
| **Acaca** | Forward | GTTCTGTTGGACAACGCCTTCAC |  |
|  | Reverse | GGAGTCACAGAAGCAGCCCATT |  |
| **Fasn** | Forward | CACAGTGCTCAAAGGACATGCC |  |
|  | Reverse | CACCAGGTGTAGTGCCTTCCTC |  |
| **Mlx** | Forward | CAGCAGGATTTCTCCATTGGCTC |  |
|  | Reverse | GCAGTGTGGATACTTCTTCCTCC |  |
| **Ogt** | Forward | GGCTATGTGAGTTCTGACTTCGG |  |
|  | Reverse | GATTGGCTTCCGCCATCACCTT |  |
| **Acly** | Forward | AGGAAGTGCCACCTCCAACAGT |  |
|  | Reverse | CGCTCATCACAGATGCTGGTCA |  |
| **Ppargc1b** | Forward | CAGCCTCAGTTCCAGAAGTCAG |  |
|  | Reverse | CACCGAAGTGAGGTGCTTATGC |  |
| **Lipe** | Forward | GCTCATCTCCTATGACCTACGG |  |
|  | Reverse | TCCGTGGATGTGAACAACCAGG |  |
| **18S** | Forward | AGGCCCTGTAATTGGAATGAGTC |  |
|  | Reverse | GCTCCCAAGATCCAACTACGAG |  |
