# Supplementary material for: Integrated causal inference, kidney transcriptomics, and experimental validation identify ChREBP (MLXIPL) as a driver of maladaptive metabolic remodeling in diabetic kidney disease
Source: Front Endocrinol (Lausanne). 2026 Apr 15;17:1809567. doi: 10.3389/fendo.2026.1809567 (PMC13125001; doi:10.3389/fendo.2026.1809567)
Supplement: Supplementary file 8 [file Table4.docx]

| id.exposure | id.outcome | outcome | exposure | method | Q | Q_df | Q_pval |
| --- | --- | --- | --- | --- | --- | --- | --- |
| eQTLGen (Blood) | finn-b-E4_DIABETES | Diabetes mellitus \|\| id:finn-b-E4_DIABETES | MLXIPL | MR Egger | 9.867844967 | 2 | 0.007198 |
| eQTLGen (Blood) | finn-b-E4_DIABETES | Diabetes mellitus \|\| id:finn-b-E4_DIABETES | MLXIPL | Inverse variance weighted | 10.03320664 | 3 | 0.018286 |
| eQTLGen (Blood) | finn-b-E4_DM2 | Type 2 diabetes \|\| id:finn-b-E4_DM2 | MLXIPL | MR Egger | 10.16695945 | 2 | 0.006198 |
| eQTLGen (Blood) | finn-b-E4_DM2 | Type 2 diabetes \|\| id:finn-b-E4_DM2 | MLXIPL | Inverse variance weighted | 10.35831867 | 3 | 0.015753 |
| eQTLGen (Blood) | finn-b-E4_DM2_STRICT | Type 2 diabetes, strict (exclude DM1) \|\| id:finn-b-E4_DM2_STRICT | MLXIPL | MR Egger | 9.089872272 | 2 | 0.010621 |
| eQTLGen (Blood) | finn-b-E4_DM2_STRICT | Type 2 diabetes, strict (exclude DM1) \|\| id:finn-b-E4_DM2_STRICT | MLXIPL | Inverse variance weighted | 9.234993511 | 3 | 0.026324 |
| eQTLGen (Blood) | finn-b-E4_DM2NASCOMP | Type 2 diabetes with other specified/multiple/unspecified complications \|\| id:finn-b-E4_DM2NASCOMP | MLXIPL | MR Egger | 7.50643481 | 2 | 0.023442 |
| eQTLGen (Blood) | finn-b-E4_DM2NASCOMP | Type 2 diabetes with other specified/multiple/unspecified complications \|\| id:finn-b-E4_DM2NASCOMP | MLXIPL | Inverse variance weighted | 7.656905738 | 3 | 0.053661 |
| eQTLGen (Blood) | finn-b-KELA_DIAB_INSUL | Diabetes, insuline treatment (Kela reimbursement) \|\| id:finn-b-KELA_DIAB_INSUL | MLXIPL | MR Egger | 10.00396429 | 2 | 0.006725 |
| eQTLGen (Blood) | finn-b-KELA_DIAB_INSUL | Diabetes, insuline treatment (Kela reimbursement) \|\| id:finn-b-KELA_DIAB_INSUL | MLXIPL | Inverse variance weighted | 10.17540817 | 3 | 0.017132 |
| eQTLGen (Blood) | finn-b-T2D | Type 2 diabetes, definitions combined \|\| id:finn-b-T2D | MLXIPL | MR Egger | 9.25018134 | 2 | 0.009803 |
| eQTLGen (Blood) | finn-b-T2D | Type 2 diabetes, definitions combined \|\| id:finn-b-T2D | MLXIPL | Inverse variance weighted | 9.394138845 | 3 | 0.024485 |
| eQTLGen (Blood) | ieu-a-1101 | Urinary albumin-to-creatinine ratio \|\| id:ieu-a-1101 | MLXIPL | Inverse variance weighted | 0.249319596 | 1 | 0.617555 |
| eQTLGen (Blood) | ieu-a-1107 | Urinary albumin-to-creatinine ratio \|\| id:ieu-a-1107 | MLXIPL | Inverse variance weighted | 0.977152347 | 1 | 0.322903 |

**TableS4. Assessment of heterogeneity among instrumental variables for Type 2 Diabetes and renal outcomes.**
