# Supplementary material for: Integrated causal inference, kidney transcriptomics, and experimental validation identify ChREBP (MLXIPL) as a driver of maladaptive metabolic remodeling in diabetic kidney disease
Source: Front Endocrinol (Lausanne). 2026 Apr 15;17:1809567. doi: 10.3389/fendo.2026.1809567 (PMC13125001; doi:10.3389/fendo.2026.1809567)
Supplement: Supplementary file 9 [file Table5.docx]

| id.exposure | id.outcome | outcome | exposure | egger_intercept | se | pval |
| --- | --- | --- | --- | --- | --- | --- |
| eQTLGen (Blood) | finn-b-E4_DIABETES | Diabetes mellitus \|\| id:finn-b-E4_DIABETES | MLXIPL | 0.026523 | 0.144878 | 0.87162 |
| eQTLGen (Blood) | finn-b-E4_DM2 | Type 2 diabetes \|\| id:finn-b-E4_DM2 | MLXIPL | 0.030087 | 0.155073 | 0.864081 |
| eQTLGen (Blood) | finn-b-E4_DM2_STRICT | Type 2 diabetes, strict (exclude DM1) \|\| id:finn-b-E4_DM2_STRICT | MLXIPL | 0.027384 | 0.153249 | 0.874643 |
| eQTLGen (Blood) | finn-b-E4_DM2NASCOMP | Type 2 diabetes with other specified/multiple/unspecified complications \|\| id:finn-b-E4_DM2NASCOMP | MLXIPL | 0.030122 | 0.150436 | 0.859816 |
| eQTLGen (Blood) | finn-b-KELA_DIAB_INSUL | Diabetes, insuline treatment (Kela reimbursement) \|\| id:finn-b-KELA_DIAB_INSUL | MLXIPL | 0.029027 | 0.156788 | 0.870197 |
| eQTLGen (Blood) | finn-b-T2D | Type 2 diabetes, definitions combined \|\| id:finn-b-T2D | MLXIPL | 0.027272 | 0.154583 | 0.876209 |
| eQTLGen (Blood) | ieu-a-1101 | Urinary albumin-to-creatinine ratio \|\| id:ieu-a-1101 | MLXIPL | NA | NA | NA |
| eQTLGen (Blood) | ieu-a-1107 | Urinary albumin-to-creatinine ratio \|\| id:ieu-a-1107 | MLXIPL | NA | NA | NA |

**TableS5.Evaluation of directional horizontal pleiotropy using MR-Egger intercept tests.**
