# Supplementary material for: Integrated causal inference, kidney transcriptomics, and experimental validation identify ChREBP (MLXIPL) as a driver of maladaptive metabolic remodeling in diabetic kidney disease
Source: Front Endocrinol (Lausanne). 2026 Apr 15;17:1809567. doi: 10.3389/fendo.2026.1809567 (PMC13125001; doi:10.3389/fendo.2026.1809567)
Supplement: Supplementary file 10 [file Table6.docx]

| model | Gene | logFC | P.Value | adj.P.Val |
| --- | --- | --- | --- | --- |
| Unadjusted | MLXIPL | -0.72271 | 0.000992 | 0.012675 |
| Adjust_TubuleIntegrity | MLXIPL | -0.54923 | 0.001862 | 0.018353 |
| Adjust_ImmuneGeneral | MLXIPL | -0.47468 | 0.106989 | 0.206643 |
| Adjust_FibrosisECM | MLXIPL | -0.42855 | 0.077995 | 0.221172 |

**TableS6. Multivariable regression analysis of *MLXIPL* differential expression in diabetic kidney disease adjusting for structural and cellular composition.**
