# Supplementary material for: Integrated causal inference, kidney transcriptomics, and experimental validation identify ChREBP (MLXIPL) as a driver of maladaptive metabolic remodeling in diabetic kidney disease
Source: Front Endocrinol (Lausanne). 2026 Apr 15;17:1809567. doi: 10.3389/fendo.2026.1809567 (PMC13125001; doi:10.3389/fendo.2026.1809567)
Supplement: Supplementary file 11 [file Table7.docx]

| Variable | rho | p | n |
| --- | --- | --- | --- |
| Tubular_Integrity | 0.498588368 | 0.019440087 | 22 |
| Fibrosis | -0.605872388 | 0.003387579 | 22 |
| Immune | -0.622811971 | 0.00243778 | 22 |
| ChREBP_Target_Score | -0.025409373 | 0.911805552 | 22 |

TableS7. Correlations between bulk-tissue MLXIPL expression and tubular integrity, fibrosis, immune, and ChREBP target signatures in GSE30529.
