# Supplementary material for: Integrated causal inference, kidney transcriptomics, and experimental validation identify ChREBP (MLXIPL) as a driver of maladaptive metabolic remodeling in diabetic kidney disease
Source: Front Endocrinol (Lausanne). 2026 Apr 15;17:1809567. doi: 10.3389/fendo.2026.1809567 (PMC13125001; doi:10.3389/fendo.2026.1809567)
Supplement: Supplementary file 12 [file Table8.docx]

| **Model** | **Covariates included** | **groupDKD β (SE)** | **P for groupDKD** | **Tubular_Integrity β (SE)** | **P** | **Fibrosis β (SE)** | **P** | **Immune β (SE)** | **P** | **Adjusted R²** |
| --- | --- | --- | --- | --- | --- | --- | --- | --- | --- | --- |
| LM1 | group only | -0.723 (0.198) | 0.0016 | — | — | — | — | — | — | 0.369 |
| LM2 | group + Tubular_Integrity | -0.577 (0.160) | 0.00185 | 0.335 (0.090) | 0.00145 | — | — | — | — | 0.616 |
| LM3 | group + Tubular_Integrity + Fibrosis | -0.544 (0.211) | 0.0191 | 0.314 (0.126) | 0.0225 | -0.048 (0.197) | 0.8116 | — | — | 0.596 |
| LM4 | group + Tubular_Integrity + Fibrosis + Immune | -0.638 (0.291) | 0.0426 | 0.333 (0.134) | 0.0241 | -0.095 (0.225) | 0.6765 | 0.138 (0.288) | 0.6378 | 0.578 |

Table S8. Multivariable linear models evaluating the association between DKD status and bulk-tissue MLXIPL expression in GSE30529.

β coefficients were derived from linear regression models with MLXIPL expression as the dependent variable. Adjusted R² values are shown for each model.
