# Supplementary material for: Integrated causal inference, kidney transcriptomics, and experimental validation identify ChREBP (MLXIPL) as a driver of maladaptive metabolic remodeling in diabetic kidney disease
Source: Front Endocrinol (Lausanne). 2026 Apr 15;17:1809567. doi: 10.3389/fendo.2026.1809567 (PMC13125001; doi:10.3389/fendo.2026.1809567)
Supplement: Supplementary file 13 [file Table9.docx]

### Table S9 GSEA enrichment analysis of high and low expression groups of target gene *MLXIPL*

| ID | setSize | enrichmentScore | NES | pvalue | p.adjust | qvalue |
| --- | --- | --- | --- | --- | --- | --- |
| REACTOME_FATTY_ACID_METABOLISM | 136 | 0.616183189 | 2.335795354 | 1E-10 | 1.99917E-08 | 1.50263E-08 |
| WP_AMINO_ACID_METABOLISM | 77 | 0.665152751 | 2.326925914 | 7.24265E-10 | 9.65285E-08 | 7.25536E-08 |
| REACTOME_MITOCHONDRIAL_FATTY_ACID_BETA_OXIDATION | 25 | 0.839800389 | 2.325054659 | 2.52365E-08 | 2.08766E-06 | 1.56915E-06 |
| REACTOME_METABOLISM_OF_AMINO_ACIDS_AND_DERIVATIVES | 287 | 0.549333987 | 2.281283232 | 1E-10 | 1.99917E-08 | 1.50263E-08 |

GSEA，Gene Set Enrichment Analysis。
