# Supplementary material for: Integrated causal inference, kidney transcriptomics, and experimental validation identify ChREBP (MLXIPL) as a driver of maladaptive metabolic remodeling in diabetic kidney disease
Source: Front Endocrinol (Lausanne). 2026 Apr 15;17:1809567. doi: 10.3389/fendo.2026.1809567 (PMC13125001; doi:10.3389/fendo.2026.1809567)
Supplement: Supplementary file 14 [file Table10.docx]

**Table S10.Results of GSVA(Low/High) for GSE30529**

| ID | logFC | AveExpr | t | P.Value | adj.P.Val | B |
| --- | --- | --- | --- | --- | --- | --- |
| HALLMARK_TGF_BETA_SIGNALING | -0.569 | -0.00301 | -3.83085 | 0.000435 | 0.021735 | -0.11644 |
| HALLMARK_G2M_CHECKPOINT | -0.49021 | -0.00752 | -3.30715 | 0.00198 | 0.049511 | -1.50671 |
| HALLMARK_E2F_TARGETS | -0.42812 | -0.04456 | -3.03463 | 0.004191 | 0.069844 | -2.1855 |
| HALLMARK_MITOTIC_SPINDLE | -0.35064 | -0.06285 | -2.77391 | 0.008332 | 0.099224 | -2.80083 |
| HALLMARK_OXIDATIVE_PHOSPHORYLATION | 0.496798 | 0.064675 | 2.705631 | 0.009922 | 0.099224 | -2.95595 |
| HALLMARK_ANGIOGENESIS | -0.36897 | -0.01305 | -2.28824 | 0.027407 | 0.22839 | -3.84387 |
| HALLMARK_ADIPOGENESIS | 0.340413 | 0.016311 | 2.119334 | 0.040229 | 0.244085 | -4.17123 |
| HALLMARK_EPITHELIAL_MESENCHYMAL_TRANSITION | -0.35887 | -0.01184 | -2.08422 | 0.043479 | 0.244085 | -4.23681 |
| HALLMARK_IL6_JAK_STAT3_SIGNALING | -0.33804 | -0.00978 | -2.03279 | 0.048653 | 0.244085 | -4.33126 |
| HALLMARK_XENOBIOTIC_METABOLISM | 0.325854 | 0.009523 | 2.031243 | 0.048817 | 0.244085 | -4.33407 |
| HALLMARK_HEDGEHOG_SIGNALING | -0.31006 | -0.00776 | -1.95271 | 0.057776 | 0.245698 | -4.4745 |
| HALLMARK_TNFA_SIGNALING_VIA_NFKB | -0.31496 | -0.01262 | -1.90845 | 0.063425 | 0.245698 | -4.55165 |
| HALLMARK_UNFOLDED_PROTEIN_RESPONSE | -0.29932 | -0.00784 | -1.90503 | 0.063881 | 0.245698 | -4.55755 |
| HALLMARK_FATTY_ACID_METABOLISM | 0.325327 | 0.036104 | 1.865654 | 0.069332 | 0.247614 | -4.62485 |
| HALLMARK_COMPLEMENT | -0.28386 | -0.0032 | -1.80382 | 0.078688 | 0.248201 | -4.72815 |
| HALLMARK_APOPTOSIS | -0.28687 | -0.00812 | -1.79922 | 0.079424 | 0.248201 | -4.73571 |
| HALLMARK_REACTIVE_OXYGEN_SPECIES_PATHWAY | 0.261715 | 0.008414 | 1.66551 | 0.103516 | 0.281964 | -4.94845 |
| HALLMARK_INFLAMMATORY_RESPONSE | -0.26656 | -0.04047 | -1.65478 | 0.105687 | 0.281964 | -4.96491 |
| HALLMARK_UV_RESPONSE_DN | -0.25387 | -0.03716 | -1.64766 | 0.107146 | 0.281964 | -4.97577 |
| HALLMARK_UV_RESPONSE_UP | 0.248271 | 0.006293 | 1.589915 | 0.119617 | 0.299043 | -5.06241 |
| HALLMARK_ALLOGRAFT_REJECTION | -0.27475 | -0.02321 | -1.52443 | 0.135159 | 0.321399 | -5.15737 |
| HALLMARK_MYOGENESIS | 0.226722 | -0.03685 | 1.498051 | 0.141861 | 0.321399 | -5.19462 |
| HALLMARK_MYC_TARGETS_V2 | 0.234186 | -0.02629 | 1.475329 | 0.147843 | 0.321399 | -5.22624 |
| HALLMARK_HEME_METABOLISM | 0.21452 | 0.031686 | 1.351626 | 0.183984 | 0.371961 | -5.39075 |
| HALLMARK_INTERFERON_GAMMA_RESPONSE | -0.24194 | -0.01358 | -1.34536 | 0.18598 | 0.371961 | -5.39873 |
| HALLMARK_IL2_STAT5_SIGNALING | -0.19141 | 0.019511 | -1.21639 | 0.230867 | 0.443976 | -5.55552 |
| HALLMARK_WNT_BETA_CATENIN_SIGNALING | -0.20084 | -0.02389 | -1.17993 | 0.244901 | 0.453521 | -5.5972 |
| HALLMARK_INTERFERON_ALPHA_RESPONSE | -0.19938 | -0.02575 | -1.12175 | 0.268565 | 0.462579 | -5.66124 |
| HALLMARK_KRAS_SIGNALING_UP | -0.18315 | -0.00384 | -1.12034 | 0.269156 | 0.462579 | -5.66275 |
| HALLMARK_BILE_ACID_METABOLISM | 0.214281 | 0.069173 | 1.100624 | 0.277548 | 0.462579 | -5.68375 |
| HALLMARK_PI3K_AKT_MTOR_SIGNALING | -0.16904 | 0.000731 | -1.05873 | 0.295987 | 0.477399 | -5.72719 |
| HALLMARK_PANCREAS_BETA_CELLS | 0.152474 | -0.02693 | 0.922051 | 0.361953 | 0.562869 | -5.85777 |
| HALLMARK_MYC_TARGETS_V1 | -0.13878 | -0.03395 | -0.90369 | 0.371494 | 0.562869 | -5.874 |
| HALLMARK_PEROXISOME | 0.149828 | 0.043888 | 0.828909 | 0.412006 | 0.590533 | -5.93686 |
| HALLMARK_DNA_REPAIR | 0.133692 | -0.00962 | 0.826467 | 0.413373 | 0.590533 | -5.93883 |
| HALLMARK_APICAL_SURFACE | -0.13839 | 0.004708 | -0.7986 | 0.429173 | 0.59112 | -5.96085 |
| HALLMARK_APICAL_JUNCTION | -0.13086 | -0.01412 | -0.78428 | 0.437429 | 0.59112 | -5.97188 |
| HALLMARK_ANDROGEN_RESPONSE | -0.13016 | 0.038344 | -0.75255 | 0.456067 | 0.600088 | -5.99564 |
| HALLMARK_CHOLESTEROL_HOMEOSTASIS | 0.109522 | 0.081971 | 0.550301 | 0.58513 | 0.750167 | -6.1245 |
| HALLMARK_GLYCOLYSIS | 0.079651 | 0.003933 | 0.510936 | 0.612165 | 0.759168 | -6.14501 |
| HALLMARK_ESTROGEN_RESPONSE_LATE | 0.077066 | -0.00818 | 0.482668 | 0.631926 | 0.759168 | -6.15881 |
| HALLMARK_ESTROGEN_RESPONSE_EARLY | -0.07479 | -0.00135 | -0.47448 | 0.637701 | 0.759168 | -6.16267 |
| HALLMARK_SPERMATOGENESIS | 0.061467 | 0.012058 | 0.404196 | 0.688192 | 0.784924 | -6.19305 |
| HALLMARK_HYPOXIA | 0.054067 | -0.05461 | 0.400714 | 0.690733 | 0.784924 | -6.19443 |
| HALLMARK_KRAS_SIGNALING_DN | 0.044556 | 0.028458 | 0.277549 | 0.782768 | 0.869742 | -6.23562 |
| HALLMARK_MTORC1_SIGNALING | -0.03338 | 0.040139 | -0.19562 | 0.845883 | 0.919438 | -6.25476 |
| HALLMARK_P53_PATHWAY | 0.017384 | -0.03934 | 0.116358 | 0.907943 | 0.965897 | -6.26698 |
| HALLMARK_PROTEIN_SECRETION | -0.01151 | 0.001085 | -0.06382 | 0.949424 | 0.983489 | -6.27166 |
| HALLMARK_NOTCH_SIGNALING | -0.00465 | 0.045626 | -0.02738 | 0.978293 | 0.983489 | -6.2733 |
| HALLMARK_COAGULATION | -0.00333 | 0.022658 | -0.02082 | 0.983489 | 0.983489 | -6.27346 |

GSVA，Gene Set Variation Analysis。
