# Supplementary material for: Integrated causal inference, kidney transcriptomics, and experimental validation identify ChREBP (MLXIPL) as a driver of maladaptive metabolic remodeling in diabetic kidney disease
Source: Front Endocrinol (Lausanne). 2026 Apr 15;17:1809567. doi: 10.3389/fendo.2026.1809567 (PMC13125001; doi:10.3389/fendo.2026.1809567)
Supplement: Supplementary file 15 [file Table11.docx]

### Table S11 Results of GO and KEGG Enrichment Analysis

| ONTOLOGY | ID | Description | GeneRatio | BgRatio | pvalue | p.adjust | qvalue |
| --- | --- | --- | --- | --- | --- | --- | --- |
| BP | GO:1901605 | alpha-amino acid metabolic process | 4/19 | 203/18800 | 4.5048E-05 | 0.022839346 | 0.014652461 |
| BP | GO:0006520 | cellular amino acid metabolic process | 4/19 | 285/18800 | 0.000167481 | 0.028304259 | 0.018158447 |
| BP | GO:0055088 | lipid homeostasis | 3/19 | 173/18800 | 0.000665801 | 0.058908406 | 0.037792375 |
| BP | GO:0046395 | carboxylic acid catabolic process | 3/19 | 238/18800 | 0.001671047 | 0.094202436 | 0.060435072 |
| BP | GO:0016054 | organic acid catabolic process | 3/19 | 242/18800 | 0.001752613 | 0.094202436 | 0.060435072 |
| MF | GO:0005543 | phospholipid binding | 3/19 | 467/18410 | 0.011615379 | 0.088661762 | 0.053330383 |
| MF | GO:0008235 | metalloexopeptidase activity | 2/19 | 72/18410 | 0.002470609 | 0.088661762 | 0.053330383 |
| MF | GO:0016209 | antioxidant activity | 2/19 | 85/18410 | 0.003423294 | 0.088661762 | 0.053330383 |
| MF | GO:0008238 | exopeptidase activity | 2/19 | 102/18410 | 0.004888054 | 0.088661762 | 0.053330383 |
| MF | GO:0016810 | hydrolase activity, acting on carbon-nitrogen (but not peptide) bonds | 2/19 | 126/18410 | 0.007363811 | 0.088661762 | 0.053330383 |

GO，Gene Ontology；BP，Biological Process；MF，Molecular Function；KEGG，Kyoto Encyclopedia of Genes and Genomes。
