# Supplementary material for: Integrated causal inference, kidney transcriptomics, and experimental validation identify ChREBP (MLXIPL) as a driver of maladaptive metabolic remodeling in diabetic kidney disease
Source: Front Endocrinol (Lausanne). 2026 Apr 15;17:1809567. doi: 10.3389/fendo.2026.1809567 (PMC13125001; doi:10.3389/fendo.2026.1809567)
Supplement: Supplementary file 16 [file Table12.docx]

### Table S12 mRNA-TF interaction network nodes.

| mRNA | TF |
| --- | --- |
| MLXIPL | CTCF |
| MLXIPL | FOS |
| MLXIPL | FOSL2 |
| MLXIPL | JUN |
| MLXIPL | JUND |
| MLXIPL | MAX |
| MLXIPL | MYC |
| MLXIPL | NRF1 |
| MLXIPL | POLR2A |
| MLXIPL | RAD21 |
| MLXIPL | REST |
| MLXIPL | SPI1 |
| MLXIPL | STAT3 |
| MLXIPL | TEAD4 |
| MLXIPL | USF1 |

TF：Transcription factors。
