# Supplementary material for: Integrated causal inference, kidney transcriptomics, and experimental validation identify ChREBP (MLXIPL) as a driver of maladaptive metabolic remodeling in diabetic kidney disease
Source: Front Endocrinol (Lausanne). 2026 Apr 15;17:1809567. doi: 10.3389/fendo.2026.1809567 (PMC13125001; doi:10.3389/fendo.2026.1809567)
Supplement: Supplementary file 17 [file Table13.docx]

### Table S13 mRNA-miRNA interaction network nodes.

| mRNA | miRNA |
| --- | --- |
| MLXIPL | hsa-miR-31-5p |
| MLXIPL | hsa-miR-192-5p |
| MLXIPL | hsa-miR-490-3p |
| MLXIPL | hsa-miR-526b-5p |
| MLXIPL | hsa-miR-665 |
| MLXIPL | hsa-miR-3690 |
