# Supplementary material for: Integrated causal inference, kidney transcriptomics, and experimental validation identify ChREBP (MLXIPL) as a driver of maladaptive metabolic remodeling in diabetic kidney disease
Source: Front Endocrinol (Lausanne). 2026 Apr 15;17:1809567. doi: 10.3389/fendo.2026.1809567 (PMC13125001; doi:10.3389/fendo.2026.1809567)
Supplement: Supplementary file 18 [file Table14.docx]

### Table S14 mRNA-RBP interaction network nodes.

| mRNA | RBP |
| --- | --- |
| MLXIPL | CSTF2T |
| MLXIPL | DROSHA |
| MLXIPL | FAM120A |
| MLXIPL | GTF2F1 |
| MLXIPL | HNRNPC |
| MLXIPL | HNRNPK |
| MLXIPL | ILF3 |
| MLXIPL | PCBP2 |
| MLXIPL | PRPF4 |
| MLXIPL | RBFOX2 |
| MLXIPL | RNPS1 |
| MLXIPL | SLTM |
| MLXIPL | SRSF7 |
| MLXIPL | SRSF9 |
| MLXIPL | U2AF1 |
| MLXIPL | U2AF2 |
