# Supplementary material for: Integrated causal inference, kidney transcriptomics, and experimental validation identify ChREBP (MLXIPL) as a driver of maladaptive metabolic remodeling in diabetic kidney disease
Source: Front Endocrinol (Lausanne). 2026 Apr 15;17:1809567. doi: 10.3389/fendo.2026.1809567 (PMC13125001; doi:10.3389/fendo.2026.1809567)
Supplement: Supplementary file 19 [file Table15.docx]

### Table S15 mRNA-Drug interaction network nodes.

| mRNA | Drug |
| --- | --- |
| MLXIPL | 6-(4-chlorophenyl)imidazo(2,1-b)(1,3)thiazole-5-carbaldehyde O-(3,4-dichlorobenzyl)oxime |
| MLXIPL | Aflatoxin B1 |
| MLXIPL | Cisplatin |
| MLXIPL | Cyclosporine |
| MLXIPL | perfluorooctane sulfonic acid |
| MLXIPL | Tobacco Smoke Pollution |
